# Forecasting the impact of diabetes mellitus on tuberculosis disease incidence and mortality in India

**DOI:** 10.7189/jogh.09.020415

**Published:** 2019-12

**Authors:** Susanne F Awad, Peijue Huangfu, Houssein H Ayoub, Fiona Pearson, Soha R Dargham, Julia A Critchley, Laith J Abu-Raddad

**Affiliations:** 1 Infectious Disease Epidemiology Group, Weill Cornell Medicine-Qatar, Cornell University, Qatar Foundation - Education City, Doha, Qatar; 2 Population Health Research Institute, St George’s, University of London, London, UK; 3 Department of Mathematics, Statistics, and Physics, Qatar University, Doha, Qatar; 4 Department of Healthcare Policy and Research, Weill Cornell Medicine, Cornell University, New York, New York, USA; 5 College of Health and Life Sciences, Hamad bin Khalifa University, Doha, Qatar; * Joint senior authors

## Abstract

**Background:**

In context of the rapidly expanding diabetes mellitus (DM) epidemic in India and slowly declining tuberculosis (TB) incidence, we aimed to estimate the past, current, and future impact of DM on TB epidemiology.

**Methods:**

An age-structured TB-DM dynamical mathematical model was developed and analyzed to assess the DM-on-TB impact. The model was calibrated using a literature review and meta-analyses. The DM-on-TB impact was analyzed using population attributable fraction metrics. Sensitivity analyses were conducted by accommodating less conservative effect sizes for the TB-DM interactions, by factoring the age-dependence of the TB-DM association, and by assuming different TB disease incidence rate trajectories.

**Results:**

In 1990, 11.4% (95% uncertainty interval (UI) = 6.3%-14.4%) of new TB disease incident cases were attributed to DM. This proportion increased to 21.9% (95% UI = 12.1%-26.4%) in 2017, and 33.3% (95% UI = 19.0%-44.1%) in 2050. Similarly, in 1990, 14.5% (95% UI = 9.5%-18.2%) of TB-related deaths were attributed to DM. This proportion increased to 28.9% (95% UI = 18.9%-34.1%) in 2017, and 42.8% (95% UI = 28.7%-53.1%) in 2050. The largest impacts originated from the effects of DM on TB disease progression and infectiousness. Sensitivity analyses suggested that the impact could be even greater.

**Conclusions:**

The burgeoning DM epidemic is predicted to become a leading driver of TB disease incidence and mortality over the coming decades. By 2050, at least one-third of TB incidence and almost half of TB mortality in India will be attributed to DM. This is likely generalizable to other Asian Pacific countries with similar TB-DM burdens. Targeting the impact of the increasing DM burden on TB control is critical to achieving the goal of TB elimination by 2050.

Although tuberculosis (TB) remains a public health concern globally, several countries are disproportionally affected by TB [1]. India harbors the largest number of individuals with TB worldwide, with at least twice as many cases as any other country [1]. In 2016, 2.8 million incident TB disease cases (27% of global TB incidence) and 435 000 TB deaths (26% of global TB deaths) were estimated in India [1].

TB disease incidence is affected by key risk factors such as diabetes mellitus (DM), HIV, under-nutrition, and smoking [2]. In 2017, 73 million Indians were living with DM at a prevalence of 8.8% (95% confidence interval (CI) = 6.7%-10.9%) [3]. India was projected to account for the highest number of DM cases globally by 2045 at 134 million cases [3]. With India burdened by both TB and DM, their synergetic relationship is a major public health concern. A number of TB-DM epidemiological studies have been conducted in this country [4-9], with recent data reporting high DM prevalence among TB patients [10].

DM increases the risk of TB infection [11] and disease [12-14], and has adverse impacts on TB treatment outcomes (eg, DM increases the risk of mortality during TB treatment, TB relapse, and possibly multi-drug resistant TB) [15-20]. Several biological mechanisms appear to explain the synergetic TB-DM association [21-33]. For example, the hypothesis that DM impairs the innate and adaptive immune responses, such as interferon-C (IFN-c), necessary to prevent the proliferation of TB, is supported by existing studies [13,28,30]. Studies showed that, compared to people with no DM, IFN-c levels were significantly reduced in people with DM [30], and that IFN-c levels were negatively associated with glycated hemoglobin levels [31].

A recent study of TB-DM interactions indicated large potential impact for DM on TB incidence including both direct (eg, DM increasing the risk of onset of TB disease) and indirect effects (eg, onward transmission of TB from people with and without DM) [34]. The study concluded that the impact of DM on TB epidemiology could be underestimated, if assessed using more conventional population attributable fraction (*PAF*) approaches such as *Levin’s formula* [35], that capture only the direct impact of DM on TB [34].

Against this background, we aimed to estimate the past, current, and future impact of DM on TB epidemiology in India using a dynamical mathematical model. A strength of this study is that it accounts for the different pathways in which DM affects TB natural history and treatment outcomes, and incorporates a detailed quantitative assessment of the effect sizes of each of the DM-on-TB effects. The study also factors the projected rise of the DM epidemic in India over the coming decades, and assesses both the direct and indirect population impacts of DM on TB. The TB-DM model was applied to India to demonstrate the utility of our approach in a country highly burdened with both diseases, however, can be implemented in additional countries.

## METHODS

We constructed an age-structured deterministic compartmental model to characterize the impact of DM on TB epidemiology in India by extending a recently developed analytical approach [34]. The model was also designed based on a recently developed conceptual framework for TB-DM interactions [34]. The model was coded and analyzed in MATLAB R2015a [36].

### Mathematical model

The model is described by a system of coupled nonlinear differential equations stratifying the Indian population by age group, TB infection status, TB infection stage, TB disease form, TB treatment status, TB recovery status, and DM status. Details of the model can be found in the Online Supplementary Documents (Appendix S1 and S2 in the **Online Supplementary Document**).

The population was stratified into 20 5-year age bands representing the age cohort 0-99 years. Upon infection, TB progression was stratified into the two stages: latent-slow TB infection (LSI) and latent-fast TB infection (LFI). TB disease was stratified into the three clinically-relevant forms: smear-positive pulmonary (SP-PTB), smear-negative pulmonary (SN-PTB), and extra-pulmonary (EP-TB) [37,38]. The proportion of individuals developing each infection and disease form was age-dependent, and only the pulmonary forms were considered infectious. Treatment was assumed to last for six-months reflecting the directly-observed treatment short-course (DOTS) therapy [39].

Individuals with DM followed a distinct TB natural history from that of non-DM individuals—TB natural history was modulated by specific effects of having concurrent DM (Figure S1 in the **Online Supplementary Document**). Based on empirical evidence, DM was assumed to affect TB natural history and treatment outcomes through 10 different pathways [34]. The effects, their definitions, their effect sizes, and the evidence supporting them are summarized in **Table 1** and discussed in Appendix S2.2 in **Online Supplementary Document**.

**Table 1:** Key assumptions for the effects of diabetes mellitus (DM) on tuberculosis (TB) natural history and treatment outcomes

Effect	Description	Effects size	Range for uncertainty analysis	Distribution used for uncertainty analysis	Sources
**Effects of DM on TB natural history (TB infection and TB disease):**					
Effect 1-Susceptibility	DM increases susceptibility to TB infection	1.50	1.0-2.2	Lognormal [40]	[11]
Effect 2-Fast progression	DM increases the proportion of TB infections entering latent-fast state as opposed to latent-slow state	Fitting parameter	-	Lognormal [40]	To fit the measured meta-analytically pooled TB-DM association of 2.00 (95% CI = 1.78-2.24) [12]
Effect 3-Reactivation	DM increases the rate of developing TB disease among those with latent TB infection	1.00 (no effect)	-	-	
Effect 4-Latent reinfection	DM increases the susceptibility to TB reinfection among those with latent-slow TB infection	1.00 (no effect)	-	-	
Effect 5-Smear positivity	DM increases the proportion of new PTB^†^ disease cases progressing to SP-PTB* as opposed to SN-PTB^$^	κ = 1.25 κ’ = 0.67	κ = 1.20-1.32 κ’ = 0.65-0.68	Normal	Estimated based on meta-analysis of existing data and Equation S1-S3 (Appendix S1.3 in **Online Supplementary Document**)
Effect 6-Disease infectiousness	DM increases the infectiousness of PTB (SP-PTB and SN-PTB) for untreated and treated TB disease cases	1.46	±25%	Uniform	Estimated based on weighted average of existing data [41-47]
Effect 7-TB mortality	DM increases the hazard of TB-related mortality for untreated and treated TB disease cases	2.11	1.76-2.51	Lognormal [40]	Estimated based on meta-analysis of existing data [20]
**Effects of DM on TB treatment outcomes:**					
Effect 8-Treatment failure	DM reduces the proportion of successful treatment (through increased risk of treatment failure and MDR-TB^¥^)	1.00 (no effect)	-	-	Estimated based on meta-analysis of existing data (Appendix S2.2 in **Online Supplementary Document**)
Effect 9-Recovery	DM reduces the rate of TB recovery (ie, prolongs the recovery time) for those who recover naturally or due to treatment	0.82	±25%	Uniform	Estimated based on weighted average of existing data [6,41,48]
Effect 10-Cured reinfection	DM increases susceptibility to TB reinfection among those treated or recovered from TB disease	1.80	1.40-2.30	Lognormal [40]	Estimated based on meta-analysis of existing data [20]

PTB – Pulmonary tuberculosis, SP-PTB – smear-positive pulmonary TB, SN-PTB –smear-negative pulmonary TB, MDR-TB – multi-drug resistant TB

Briefly, compared to non-DM individuals, DM increased susceptibility to TB infection (*Effect 1-Susceptibility*), proportion of TB infections entering LFI vs LSI states (*Effect 2-Fast progression*), proportion of those developing SP-PTB (vs SN-PTB) for those with pulmonary TB disease (*Effect 5-Smear positivity*), and TB infectiousness among those with pulmonary TB disease (*Effect 6-Disease infectiousness*). Furthermore, compared to non-DM individuals, DM increased the risk of TB-related mortality (*Effect 7-TB mortality*), reduced the proportion of successful treatment among those undergoing TB treatment (*Effect 8-Treatment failure*), delayed the resolution of TB disease (*Effect 9-Recovery*), and increased susceptibility to TB reinfection after recovery (*Effect 10-Cured reinfection*).

Amongst those with DM comparative to without, susceptibility to develop TB disease among those with LSI (*Effect 3-Reactivation*), and susceptibility to TB reinfection among those with LSI (*Effect 4-Primary reinfection*), were set as having no effect, as the impacts of these pathways were captured by *Effect 2*–*Fast progression* (Appendix S2.2 in the **Online Supplementary Document**). Also, given heterogeneity of evidence [20], the proportion of successful treatment among those with DM undergoing TB treatment (*Effect 8-Treatment failure*) was set as equal to those without DM undergoing TB treatment (Appendix S2.2 in **Online Supplementary Document**).

### Data sources and model fitting

TB natural history model parameters (in absence of DM) were based on available empirical evidence [37], or through model fitting to empirical data. Table S1 in **Online Supplementary Document** lists the parameter values and their sources.

The key assumptions for the effect sizes of the 10 DM-on-TB effects were based mostly on pooled evidence from systematic reviews and/or meta-analyses, or derived from specific observational studies (**Table 1** and Appendix S2.2 in **Online Supplementary Document**). Given heterogeneities and uncertainties around the exact effect sizes, we opted for a conservative approach whereby each effect size was modest, or set at the null value if the evidence is conflicting or not firmly established (ie, DM has no effect on TB). For example, the effect size for *Effect 2-Fast progression* was set as derived using an effect size of only 2.00 for the TB-DM association – based on a conservative meta-analysis that pooled studies of different study designs (Appendix S2.2 in **Online Supplementary Document**) [12]. The effect size for *Effect 7-TB mortality* was based on a recent meta-analysis estimating a pooled mean crude odds ratio (OR) of 2.11 across 48 studies [20]. Despite evidence suggesting that previous TB disease could increase the risk of developing DM [49], we opted not to account for this bi-directionality given that current evidence is not yet conclusive for this effect. Therefore, our estimates for the impact of the TB-DM interactions on TB epidemiology are more likely to underestimate the impact, rather than overestimate it.

The model was fitted using the following India-specific data: TB-incidence and mortality rates as reported in the World Health Organization (WHO) Global Health Observatory data repository [50], national and age-specific DM prevalence as reported by the International Diabetes Federation [3,51-55], age-specific DM prevalence distribution as reported by the nationally-representative Indian Council of Medical Research-India Diabetes study [56], and demographics as reported in the database of the Population Division of the United Nations Department of Economic and Social Affairs [57]. TB contact and case-detection rates were derived by model fitting to the above data.

### TB-DM synergy metric

We estimated the impact of DM on each of TB disease incidence and mortality between 1990 and 2050 by calculating the “true” *PAF* [34], ie, the proportion of each of TB incidence and mortality that is *directly* (etiologically) and *indirectly* (such as onward transmission) attributed to DM (Appendix S3 in **Online Supplementary Document**). In contrast with *Levin’s PAF* [35] which only estimates the direct population impact of DM on TB disease, “true” *PAF* (below noted only as *PAF*) was estimated for each of TB incidence and mortality as the proportional reduction between the measures in a scenario where the synergy in the TB-DM relationship is active, compared to a scenario where the synergy is inactive. We assessed the impact of DM on TB epidemiology for each of the DM effects in combinations and individually.

### Uncertainty analysis

A multivariate uncertainty analysis was conducted factoring the uncertainty in our knowledge of the DM-on-TB effect sizes (**Table 1**). We used Monte Carlo sampling from either the CI for the TB-DM effect sizes, or assuming (if uncertainty is not captured by CI) ±25% uncertainty around the point estimates for the effect sizes. We implemented 500 uncertainty runs of the model. In each run, the values of the effect sizes were randomly selected from their specified ranges, and the model was refitted to India’s country-specific data. The mean and 95% uncertainty intervals (UI) for the *PAF*s were derived from the likelihood distribution generated by the uncertainty runs.

### Sensitivity analyses

Given that our main estimates were generated using a conservative approach, we conducted two sensitivity analyses with less conservative effect sizes for the TB-DM interactions. In the first sensitivity analysis, we used, for *Effect 2-Fast progression*, the TB-DM association effect size of 3.59 based on the prospective cohort studies (Appendix S2.2 in **Online Supplementary Document**) [12], In the second sensitivity analysis, we used, for *Effect 7-TB mortality*, the effect size of 4.95 based on the pooled analysis that included studies that appropriately adjusted for confounders Appendix S2.2 in **Online Supplementary Document****)** [20].

In a third sensitivity analysis, we explored the TB-DM synergy implications by factoring the age-dependence of the TB-DM association, based on a cohort study that estimated the age-specific relative risks (RRs) of the effect of DM on TB disease [58]. In doing so, we scaled down (conservatively) the age effects reported by Kim et al [58], to reach the assumed 2-fold overall RR (Appendix S2.2 in **Online Supplementary Document**).

In a fourth sensitivity analysis, in context of uncertainty about the future trajectory of the TB epidemic over the coming decades, we assessed the TB-DM synergy implications assuming 10 different TB disease incidence rate trajectories over the coming decades. The change in TB incidence rate at 2050, relative to the baseline model scenario, was assumed to range between ±50%.

In a fifth sensitivity analysis, we accounted for the age-dependency in the proportion of individuals developing each infection form (LSI vs LFI) for those aged 15 years and above, compared with the baseline analysis in which this proportion did not differ by age for adults. Specifically, as informed by evidence [59], we assessed the TB-DM synergy implications assuming that 25% of individuals who progress to TB infection aged 15-35 years develop LFI, while only 5% of individuals aged 35+ years develop LFI.

In a sixth sensitivity analysis, we assessed the implications of assuming different TB reinfection risks [60,61]. We compared a 65% fractional reduction in the susceptibility to TB reinfection (compared to initial TB infection risk; our baseline assumption, Table S1 in **Online Supplementary Document**) [62,63], to no reduction, and to a 35% fractional increase. The different risks of reinfection were assumed for 1) individuals with LSI (that is those in latent infection), 2) individuals who successfully completed TB treatment, or 3) both individuals with LSI and those who successfully completed TB treatment.

Finally, additional sensitivity analyses were conducted to assess the sensitivity of model predictions to variations in the effect sizes of the DM-on-TB effects (**Table 1**). For each individual effect, we used the lower and upper values from either the CI for the TB-DM effect sizes, or assuming (if uncertainty is not captured by CI) ±25% uncertainty around the point estimates.

## RESULTS

**Figure 1:**
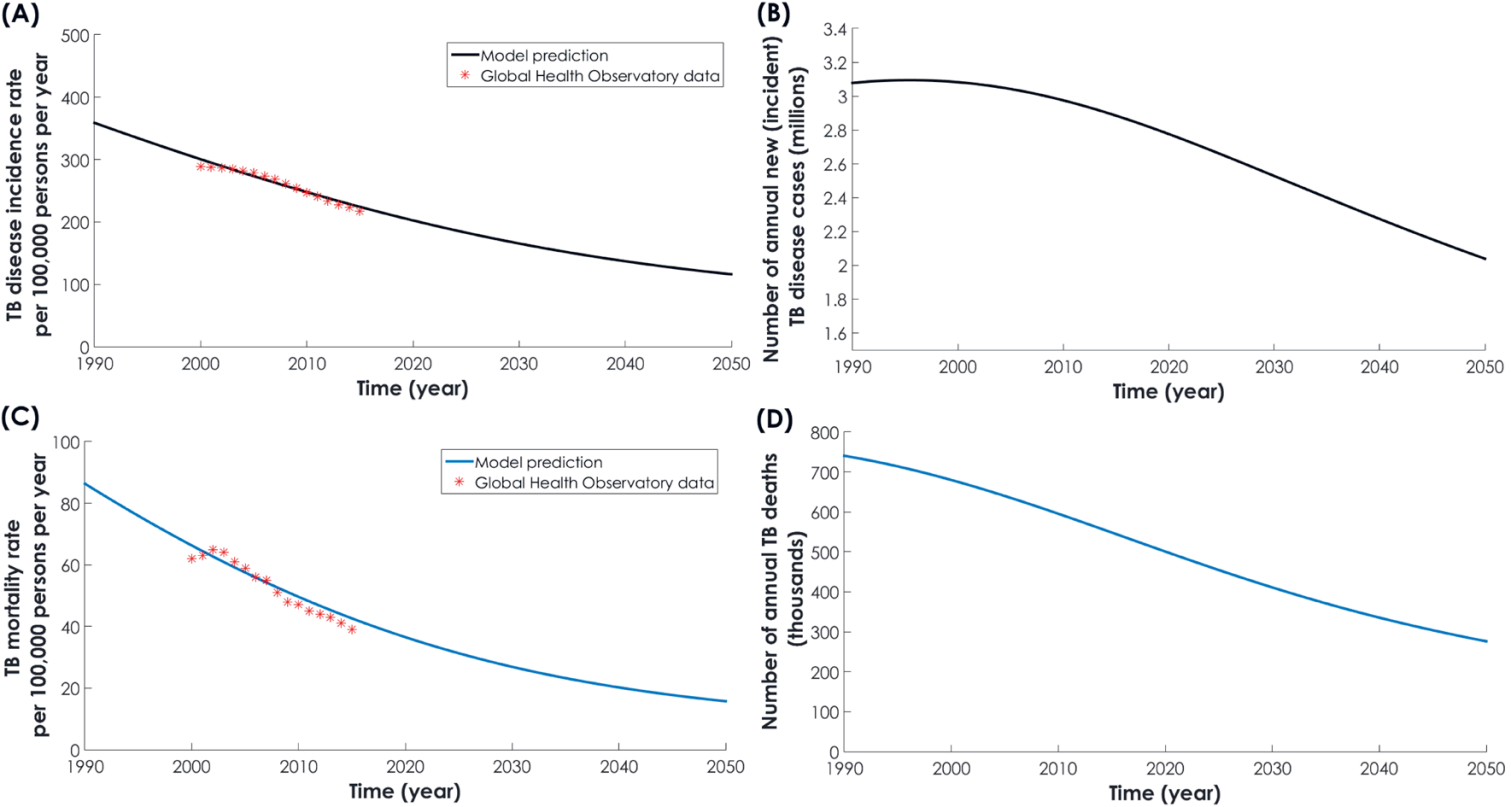
Model projections. **Panel A.** Tuberculosis (TB) disease incidence rate. **Panel B.** Number of annual new (incident) TB disease cases. **Panel C.** TB mortality rate. **Panel D.** Number of annual TB deaths, in India between 1990 and 2050. The red asterisks in panels A and C are the data provided by the World Health Organization’s Global Health Observatory data repository [50].

**Figure 2:**
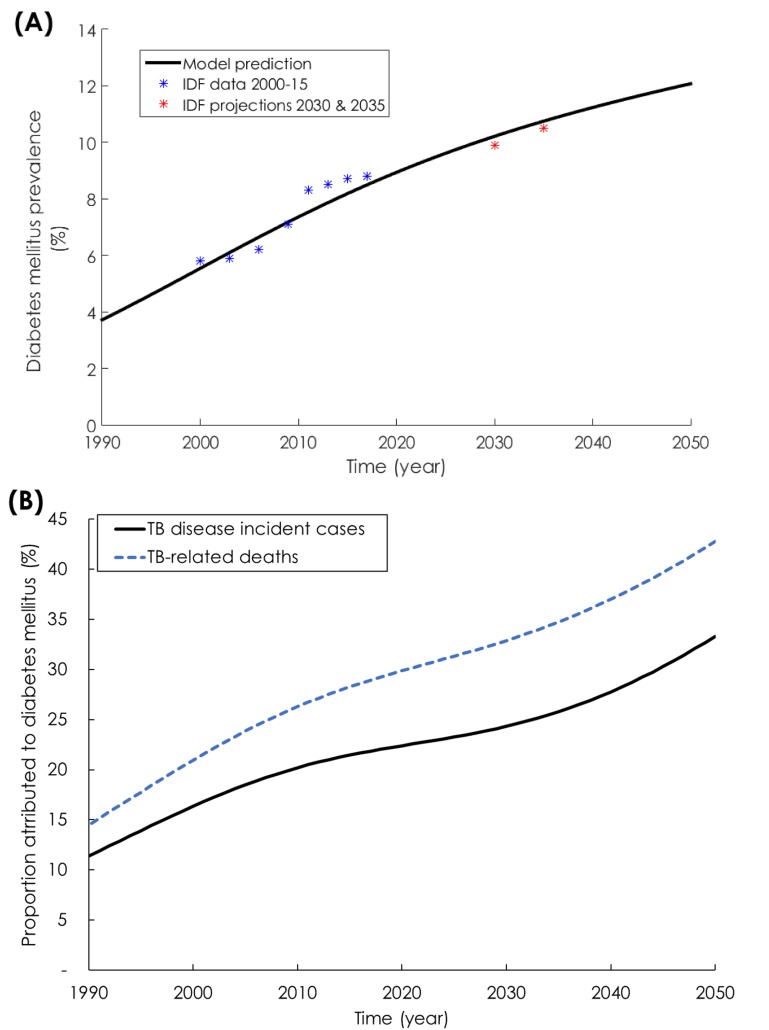
Diabetes mellitus (DM) and its projected impact on tuberculosis (TB). **Panel A.** Model projections for DM prevalence in India between 1990 and 2050. **Panel B.** Model predictions for the proportion of TB disease incident (solid black line) and mortality (dashed blue line) cases attributed to DM in India between 1990 and 2050. The blue and red asterisks in Panel A are DM prevalence data provided by the International Diabetes Federation (IDF) [53].

The model fitted well the demographic (Figure S4 in **Online Supplementary Document**), TB incidence rate (**Figure 1****,** Panel A), TB mortality rate (**Figure 1****,** Panel C), and DM prevalence data for India (**Figure 2**, Panel A). From 2017 to 2050, TB disease incidence rate (defined as the ratio of total annual number of TB disease cases over total Indian population) was projected to decrease from 215 to 116 per 100 000 persons per year (**Figure 1****,** Panel A). Meanwhile, the number of annual new (incident) cases was projected to decrease from 2.8 to 2.0 million (**Figure 1**, Panel B). Likewise, TB mortality rate (defined as the ratio of total annual number of TB-related deaths over total Indian population) was projected to decrease from 40.7 to 15.7 per 100 000 persons per year (**Figure 1****,** Panel C). Meanwhile, the number of annual TB deaths was projected to decrease from 534 000 to 287 000 (**Figure 1**, Panel D). DM prevalence in India was projected to increase from 8.5% in 2017 to 12.1% in 2050 (**Figure 2****,** Panel A).

While DM prevalence increased (**Figure 2**, Panel A) and TB incidence rate decreased (**Figure 1**, Panel A), the proportion of new TB incidence cases and proportion of TB-related deaths attributed to DM increased steadily (**Figure 2**, Panel B). In 1990, 11.4% (95% UI = 6.3%-14.4%) of new TB disease incident cases were attributed to DM (**Figure 2**, Panel B). This proportion increased to 21.9% (95% UI = 12.1%-26.4%) in 2017, and was predicted to continue increasing to 33.3% (95% UI = 19.0%-44.1%) by 2050. Similarly, in 1990, 14.5% (95% UI = 9.5%-18.2%) of TB-related deaths were attributed to DM. This proportion increased to 28.9% (95% UI = 18.9%-34.1%) in 2017, and was predicted to continue increasing to 42.8% (95% UI = 28.7%-53.1%) by 2050.

**Figure 3:**
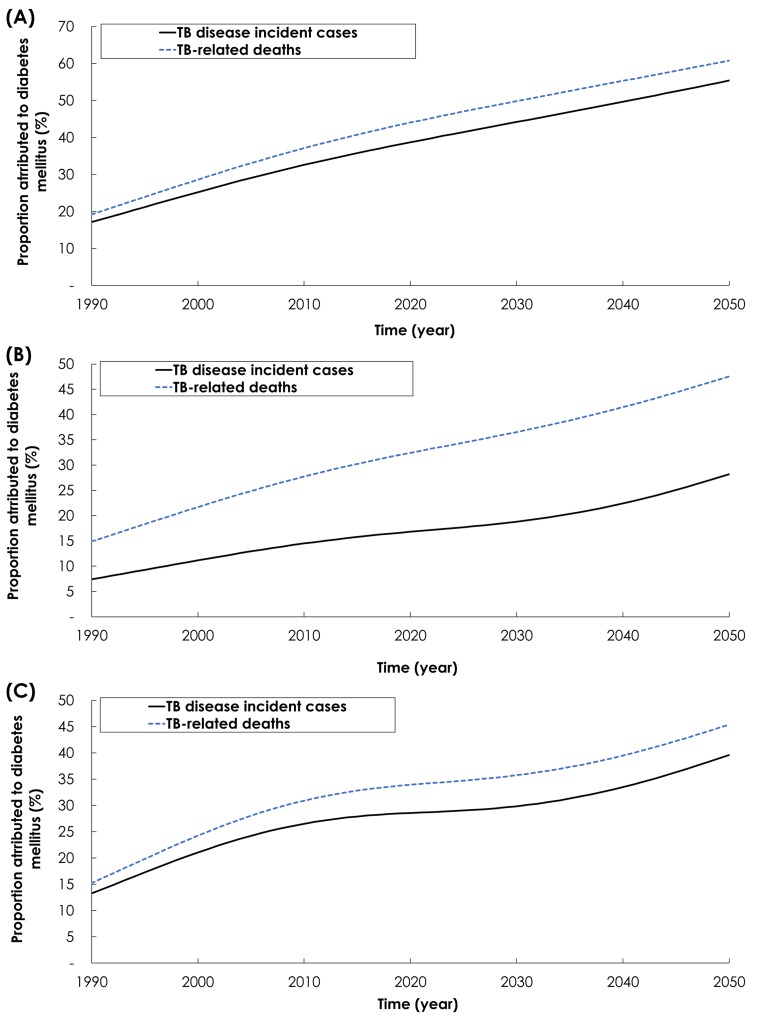
Sensitivity analyses. Model predictions for the proportion of tuberculosis (TB) disease incident (solid black line) and mortality (dashed blue line) cases attributed to DM in India between 1990 and 2050 assuming: **Panel A.** TB-DM association effect size of 3.59 based on pooling the data only from the prospective cohort studies (*Effect 2-Fast progression*, Appendix S2.2 in **Online Supplementary Document** [12]. **Panel B.** *Effect 7-TB mortality* effect size of 4.95 based on the pooled analysis that included only studies that appropriately adjusted for confounders (Appendix S2.2 in **Online Supplementary Document**) [15]. **Panel C.** Age-dependence in the TB-DM association based on a cohort study that estimated the age-specific relative risks of the effect of DM on TB disease (*Effect 2-Fast progression*, Appendix S2.2 in **Online Supplementary Document****)** [58].

Relaxing the conservative approach by using, for *Effect 2-Fast progression*, the TB-DM association effect size of 3.59 [12], resulted in a larger impact for the TB-DM synergy on TB disease incidence and mortality (**Figure 3****,** Panel A). In 1990, 17.2% of TB disease incident cases were attributed to DM, and this proportion increased to 37.0% by 2017 and 55.4% by 2050. Meanwhile, in 1990, 19.2% of TB-related deaths were attributed to DM, and this proportion increased to 42.1% by 2017 and 60.8% by 2050.

Relaxing the conservative approach by using, for *Effect 7-TB mortality*, the effect size of 4.95 [20], resulted in a larger impact for the TB-DM synergy on TB mortality but slightly smaller impact on TB disease incidence (**Figure 3**, Panel B). In 1990, 7.4% of new TB incident cases were attributed to DM, and this proportion increased to 16.2% by 2017 and 28.2% by 2050. Meanwhile, in 1990, 14.9% of TB-related deaths were attributed to DM, and this proportion increased to 31.2% by 2017 and 47.5% by 2050.

Exploring the TB-DM synergy implications by factoring the age-dependence of the TB-DM association, resulted in a larger impact on TB disease incidence and mortality (**Figure 3**, Panel C). In 1990, 13.2% of new TB incident cases were attributed to DM, and this proportion increased to 27.9% by 2017 and 39.2% by 2050. Meanwhile, in 1990, 15.3% of TB-related deaths were attributed to DM, and this proportion increased to 33.3% by 2017 and 45.41% by 2050.

Assessing the TB-DM synergy implications at different TB disease incidence trajectories over the coming decades resulted in minimal changes in the assessed impact of DM on TB incidence and mortality (Figure S5 in **Online Supplementary Document**). In 2050, new TB incident cases attributed to DM ranged between 26.5% and 34.5%, and TB-related deaths attributed to DM ranged between 37.2% and 43.7%.

Factoring the age-dependency in the proportion of individuals developing each infection form (LSI vs LFI) for those aged 15 years and above, the impact of DM on TB disease incidence and mortality was reduced (Figure S6 in **Online Supplementary Document**). In 1990, only 6.2% of new TB incident cases were attributed to DM, and this proportion increased to 12.6% by 2017 and 20.4% by 2050. Meanwhile, in 1990, 8.2% of TB-related deaths were attributed to DM, and this proportion increased to 17.7% by 2017 and 28.6% by 2050.

Exploring the TB-DM synergy implications assuming no change in the susceptibility to TB reinfection, resulted in slightly larger impact for DM on TB disease incidence and mortality (Figure S7 in **Online Supplementary Document**). By 2050, assuming no change in the susceptibility to TB reinfection among individuals who successfully completed TB treatment, with LSI, and both with LSI and those who successfully completed TB treatment, new TB incident cases attributed to DM were 33.8%, 38.6%, and 38.8%, respectively, and TB-related deaths attributed to DM were 42.1%, 44.9%, and 45.7%, respectively (Figure S7 in **Online Supplementary Document**). Exploring the TB-DM synergy implications assuming a 35% increase in the susceptibility to TB for reinfection, resulted in a relatively larger impact for DM on TB disease incidence and mortality (Figure S7 in **Online Supplementary Document**). By 2050, assuming 35% increase in the susceptibility to TB reinfection among individuals who successfully completed TB treatment, with LSI, and both with LSI and those who successfully completed TB treatment, new TB incident cases attributed to DM were 33.5%, 47.7%, and 48.9%, respectively, and TB-related deaths attributed to DM were 42.6%, 54.3%, and 57.1%, respectively (Figure S7 in **Online Supplementary Document**).

**Table 2** and **Table 3** show the individual impact of each of the DM-on-TB effects at six different time points. Most effects resulted in a larger TB disease incidence and mortality, as DM prevalence increased with time. The largest impact for TB incidence was for *Effect 2-Fast progression* followed by *Effect 6-Infectiousness* (**Table 2**). The proportion of TB incidence attributed to *Effect 2-Fast progression* increased from 8.7% in 1990 to 25.1% by 2050. The proportion of TB incidence attributed to *Effect 6-Disease infectiousness* increased from 4.5% in 1990 to 14.8% by 2050. The largest impact for TB mortality was also for *Effect 2-Fast progression* followed by *Effect 6-Infectiousness* (**Table 3**). The proportion of TB-related deaths attributed to *Effect 2-Fast progression* increased from 9.9% in 1990 to 28.5% by 2050. The proportion of TB-related deaths attributed to *Effect 6-Disease infectiousness* increased from 4.3% in 1990 to 14.4% by 2050.

**Table 2:** The epidemiologic impact of each of the individual diabetes mellitus (DM) effects on tuberculosis (TB) natural history and treatment outcomes on TB disease incidence as measured by the population attributable fraction

Time (year)	TB disease incident cases	DM prevalence (%)	Population attributable fraction (%)							
***Effect 1-Susceptibility***	***Effect 2-Fast progression***	***Effect 5-Smear positivity^†^***	***Effect 6-Disease infectiousness***	Effect 7-TB mortality*	***Effect 9-Recovery***	***Effect 10-Cured reinfection^†^***	**All effects**
1990	**3 077 706**	**3.7**	1.5	8.7	0.9	4.5	2.0	0.2	0.9	11.4
2010	**2 974 690**	**7.3**	3.1	15.5	1.2	8.2	3.2	0.4	1.7	20.2
2020	**2 775 774**	**8.9**	4.1	17.0	1.0	9.2	3.1	0.5	1.7	22.4
2030	**2 528 050**	**10.2**	5.6	18.3	0.5	10.2	3.0	0.5	1.6	24.3
2040	**2 274 153**	**11.2**	7.7	20.8	0.2	11.9	3.4	0.6	1.4	27.8
2050	**2 038 877**	**12.1**	10.8	25.1	1.1	14.8	4.2	0.8	1.5	33.3

*The impact of *Effect 7-TB mortality* on TB incidence is negative due to the fact that *Effect 7-TB mortality* reduced TB disease incidence due to the premature death of persons with TB disease.

†The impact of *Effect 5-Smear positivity* and *Effect 10-Cured reinfection* on TB incidence changed in direction with time as a consequence of a complex interplay between TB enhanced transmission, premature death of persons with TB disease, and demographic factors relating to DM age-specific prevalence distribution and TB exposure risk variation in successive birth cohorts.

**Table 3:** The epidemiologic impact of each of the individual diabetes mellitus (DM) effects on tuberculosis (TB) natural history and treatment outcomes on TB-related deaths as measured by the population attributable fraction

Time (year)	TB-related deaths	DM prevalence (%)	Population attributable fraction (%)							
***Effect 1-Susceptibility***	***Effect 2-Fast progression***	Effect 5-Smear positivity*	***Effect 6-Disease infectiousness***	***Effect 7-TB mortality***	***Effect 9-Recovery***	***Effect 10-Cured reinfection†***	**All effects**
1990	**802 790**	**3.7**	1.7	9.9	0.8	4.3	2.1	0.8	1.0	14.5
2010	**586 316**	**7.3**	3.6	18.0	1.0	8.0	4.7	1.7	1.9	26.3
2020	**491 094**	**8.9**	4.8	20.0	0.5	9.0	6.4	2.2	2.0	29.9
2030	**412 743**	**10.2**	6.6	21.5	0.4	9.9	8.1	2.6	1.8	32.9
2040	**350 711**	**11.2**	9.0	24.1	1.7	11.6	9.4	3.1	1.7	37.0
2050	**302 349**	**12.1**	12.4	28.5	3.3	14.4	10.3	3.7	1.7	42.7

*The impact of *Effect 5-Smear positivity* and *Effect 10-Cured reinfection* on TB mortality changed in direction with time as a consequence of a complex interplay between TB enhanced transmission, premature death of persons with TB disease, and demographic factors relating to DM age-specific prevalence distribution and TB exposure risk variation in successive birth cohorts.

*Effect 7-TB mortality* increased TB-related deaths from 2.1% in 1990 to 10.3% by 2050, but it reduced TB disease incidence with less TB transmission (due to the premature death of persons with TB disease). The impact of *Effect 5-Smear positivity* and *Effect 10-Cured reinfection* on both TB incidence and mortality changed in direction with time—a consequence of a complex interplay between TB enhanced transmission, premature death of TB disease cases, and demographic factors relating to DM age-specific prevalence distribution and TB exposure risk variation in successive birth cohorts.

## DISCUSSION

We provided a comprehensive quantitative assessment of the impact of DM on TB epidemiology in India, a country heavily burdened by both diseases. Anchored on a solid foundation of current empirical evidence, the assessment accounted for both direct and indirect impacts, and factored the different effects by which DM can affect TB natural history and treatment outcomes. As DM prevalence increased and TB disease incidence declined, DM was predicted to play a major and growing role in TB epidemiology. While in 1990 only one in 10 TB disease cases was attributed to DM, currently one in five is attributed to DM, and by 2050, one in three will be attributed to DM. While in 1990 only one in seven TB-related deaths was attributed to DM, currently nearly one in three is attributed to DM, and by 2050, nearly one in two will be attributed to DM. These findings highlight how DM could be emerging as the leading driver of TB incidence and mortality in India, and likely elsewhere.

The results support growing evidence highlighting the increasing role of DM on TB epidemiology [2,64,65], but also suggest that DM impact could be underestimated. We investigated DM role using a conservative approach whereby the effect size for each DM-on-TB effect was set at its lowest or null value. Setting effect sizes based on best quality evidence, resulted in even larger impact of DM on TB, particularly so for TB mortality – half of TB disease cases and TB-related deaths could be attributed to DM by 2050 (**Figure 3**).

Although the clinical effects of DM on TB treatment outcomes have been widely discussed and researched [20], the population impact has been less investigated but shown in this study to play an influential role (such as that of *Effect 7-TB mortality*). However, most of the impact of DM on TB was driven by the effects of DM on TB natural history – in particular *Effect 2-Fast progression* and *Effect 6-Disease infectiousness* (**Table 2** and **Table 3**). These findings suggest that intervention strategies should target DM patients before onset of TB disease. The population-level impacts of different intervention strategies, such as screening, case-finding, and intensified treatment, need to be investigated factoring the different DM-on-TB effects.

Our findings demonstrate that substantial reductions in TB disease incidence and mortality in India, and likely in the countries burdened by both TB and DM, are difficult to achieve without focusing on the high-level determinants and risk factors for TB including DM, as stressed in the WHO’s post-2015 TB strategy [66] and in *The Collaborative Framework for Care and Control of Tuberculosis and Diabetes* launched in 2011 by the WHO and International Union Against Tuberculosis and Lung Disease (The Union) [16], and as reinforced and expanded by the joint Union’s and World Diabetes Foundation’s “2014 Call for Action” [67] and the TB-DM Bali Declaration in 2015 [68]. Indeed, only a country-by-country approach, following the concept of “know your epidemic” for managing TB, may advance TB efforts towards TB elimination by 2050. While historically TB has been a general population infection and disease, its epidemiology could be transitioning into a new era driven by the dynamics of this infection in high risk populations such people living with DM.

Our study has limitations mostly related to incomplete knowledge of the TB-DM epidemiology. We included different DM-on-TB effects based on extensive literature review and meta-analyses of existing data (Appendix S2.2 in **Online Supplementary Document**), but we may have overlooked effects not yet supported by evidence. For example, *Effect 6-Disease infectiousness* is an effect that has not been directly investigated in the literature, but seems to have a major population-level impact on TB epidemiology through its effect on the onward transmission of the infection. The parametrization of *Effect 6-Disease infectiousness* was based on biologically-motivated plausible assumptions that need to be investigated in detail through further epidemiological/biological studies.

Evidence suggests heterogeneities and uncertainties around the exact effect sizes of several effects. For example, not all risk estimates were available by age strata, though age could be an important factor in determining the population impact of DM on TB. Moreover, even though evidence supports an increased risk of developing TB disease for those with DM [12], it does not differentiate the precise biological mechanism(s) of whether DM is acting through *Effect 2-Fast progression, Effect 3-Reactivation,* and/or *Effect 4-Primary reinfection*.

Our conclusion is predicated upon the assumption that the effect of DM on TB is causal. While strongly plausible, the scale of TB-DM biological/epidemiological synergy is not completely certain. The association could be affected by confounders (such as smoking and obesity), which are not controlled for given the very complex overlap and interactions between TB and DM. For example, the TB-DM interaction is paradoxical; while DM is known to be associated with obesity [69], TB is reportedly associated with low body mass index (ie, obesity is a protective factor against TB disease) [70]. However, when obesity and DM (a serious metabolic disorder) coexist, the protective effect of obesity on TB is attenuated [71].

We did not include all factors that may influence the impact of DM on TB, or the factors that may affect directly each of TB or DM burdens individually [12,17,72,73]. For example, the impact of HIV as a co-factor [17,72,73] was not incorporated. However, despite the potential public health implications, HIV prevalence is relatively low in India at less than 1.0% [74], hence, probably minimally affecting our results and conclusions.

We modeled TB’s natural history and dynamics based on the canonical approach in the literature [37,75], but TB’s complex natural history remains insufficiently-understood [59]. For instance, based on studies by Heimbeck [62,63], we assumed a proportional reduction in the susceptibility to TB reinfection with prior TB exposure (ie, acquired protective immunity), however, this immunity may be explained by selection bias as these studies were conducted among individuals who may not have been representative of the wider population [61]. Other evidence suggests a higher risk of reinfection rather than protective immunity [60]. Moreover, though we assumed that the proportion of individuals developing LSI vs LFI was age dependent, this was assumed for only children vs adults, but the variable age dependence could also affect the adult population [59].

We did not factor the effect of intermediate hyperglycemia (pre-DM) on TB, which may enhance the impact of DM on TB [12,76]. We only included the DM-on-TB effects, but the links between the two diseases could be bi-directional [49]. Last but not least, the impact of DM on TB depends on the trajectory of the TB epidemic over the coming decades, but this trajectory may change substantially with roll-out and scale-up of interventions in upcoming years [1].

Despite these limitations, our study has several strengths. Our model includes ten different effects in which DM affects TB natural history and treatment outcomes, incorporates a detailed quantitative assessment of the effect sizes for each effect, is age stratified to reflect the age-specific trends, and assesses both the direct and indirect population impacts of DM on TB. In addition, most of the potential limitations are likely to lead to underestimation rather than overestimation of the impact of DM on TB.

We also conducted sensitivity analyses to explore the potential impact of several mentioned limitations, and these analyses confirmed our results, or suggested that the impact could be underestimated (**Figure 3** and Figures S5 and S7 in the **Online Supplementary Document**), or slightly overestimated (Figure S6 in **Online Document**). Furthermore, our sensitivity analyses demonstrated that our results are most sensitive to *Effect 2-Fast progression, Effect 6-Disease infectiousness*, and *Effect 1-Susceptibility* (Figure S9 in **Online Supplementary Document**), as expected given the impact of these effects on TB-epidemiology (**Table 2** and **Table 3**). Otherwise, our results were largely insensitive to variations in the rest of explored effects (Figure S9 in **Online Supplementary Document**). We further conducted a multivariate uncertainty analysis by factoring the uncertainty in model parameters, and the uncertainty intervals of the model outcomes affirmed the validity of our predictions (Figure S8 in **Online Supplementary Document**). Finally, the aim of the present analysis was to assess the epidemiological implications of the TB-DM interactions focusing on the core interaction effects and at the national level. Thus, we resorted to a parsimonious model structure presenting “average” impact estimates of DM on TB, rather than stratified estimates for specific population strata.

In conclusion, the burgeoning DM epidemic in India is predicted to become a leading driver of TB disease incidence and mortality over the coming decades in India and possibly elsewhere. At present, one in five TB disease cases is attributed to DM, and by 2050, one in three will be attributed to DM. Nearly one in three TB-related deaths is attributed to DM currently, and by 2050, nearly one in two will be attributed to DM. The slowly declining TB incidence, in context of rapidly expanding DM epidemic in multiple countries, could be driving a major turn in TB epidemiology. Targeting the impact of the increasing DM burden on TB control is critical to achieving the goal of TB elimination by 2050.

## Additional material

Online Supplementary Document
