# Supplementary material for: Forecasting the impact of diabetes mellitus on tuberculosis disease incidence and mortality in India
Source: J Glob Health. 2019 Oct 22;9(2):020415. doi: 10.7189/jogh.09.020415 (PMC6815875; doi:10.7189/jogh.09.020415)
Supplement: Online Supplementary Document [file jogh-09-020415-s001.pdf]

Online Supplementary Documents

Forecasting the Impact of Diabetes Mellitus on Tuberculosis Disease Incidence and Mortality in India

Susanne F. Awad,[1,2] Peijue Huangfu,[2] Houssein H. Ayoub,[1,3,4] Fiona Pearson,[2] Soha R. Dargham,[1] Julia A. Critchley,[2] and Laith J. Abu-Raddad,[1,4,5]

[1]*Infectious Disease Epidemiology Group, Weill Cornell Medicine-Qatar, Cornell University, Qatar Foundation - Education City, Doha, Qatar*

[2]*Population Health Research Institute, St George's, University of London, London, UK*

[3]*Department of Mathematics, Statistics, and Physics, Qatar University, Doha, Qatar*

[4]*Department of Healthcare Policy and Research, Weill Cornell Medicine, Cornell University, New York, New York, USA*

[5]*College of Health and Life Sciences, Hamad bin Khalifa University, Doha, Qatar*

S1. DETAILED DESCRIPTION OF THE TB-DM MATHEMATICAL MODEL

We extended an earlier population-level deterministic mathematical model of the dynamics of tuberculosis-diabetes mellitus (TB-DM) interactions [1]. The model was described by sets of coupled nonlinear ordinary differential equations (Section S1.3) coded in MATLAB 2015a [2].

We stratified the population of India into compartments according to five-year age groups (indexed $a = 1, 2, ..., 20$ representing the 0—99 age cohort), DM status, and TB progression states. We described TB natural history (for those with and without DM) by the progression states of susceptible, latent TB infection, TB disease, treated TB disease, and recovered (Figure S1).

Figure S1. A schematic diagram of the TB-DM model. The black and red lines indicate different TB natural histories depending on DM status. The blue box/line indicates the potential TB effect on DM.

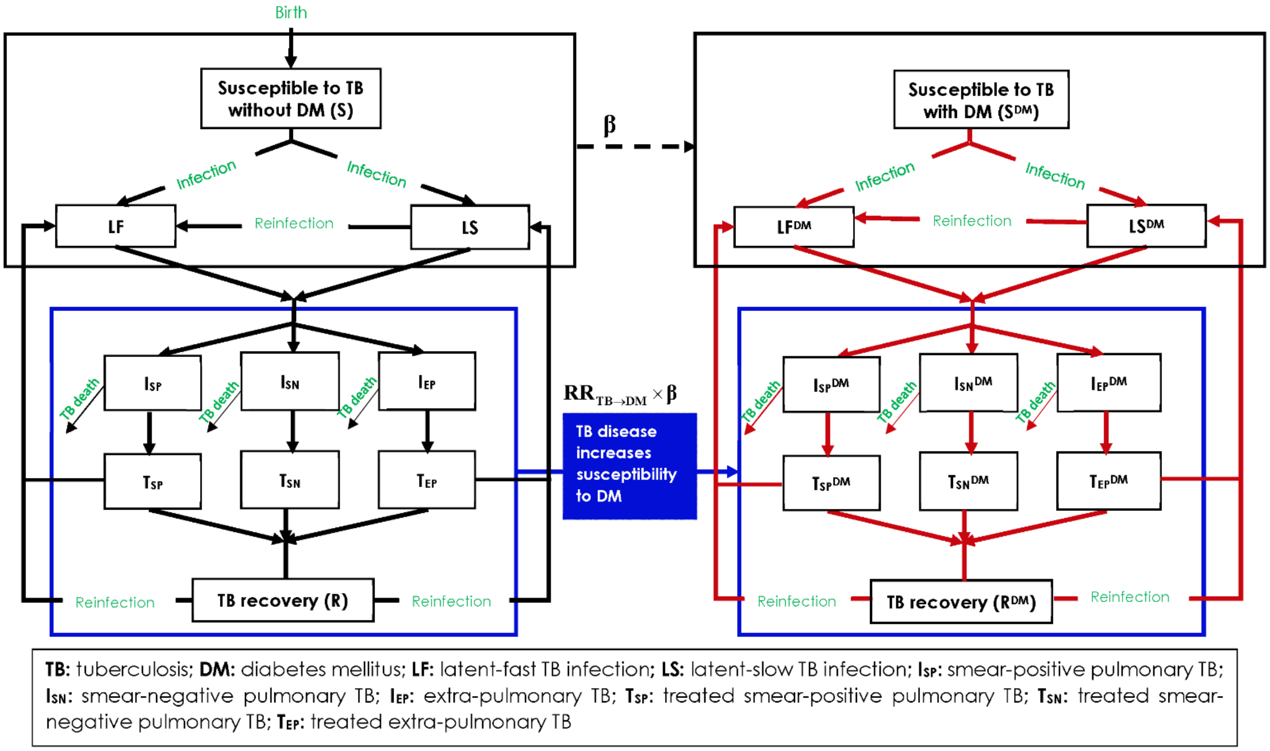

S1.1. TB transmission dynamics in absence of DM

All individuals were born ($\delta(a)\varphi(t)N_{total}$; here δ is equal one for $a=1$ and zero otherwise) susceptible to TB and DM (S), and aged at a transition rate η (i.e. from one age group to the next age group). In absence of DM, all individuals were at risk of developing DM at a rate $\beta(t,a)$ (except those 0-4 years old; $a=1$), and at risk of natural mortality at a rate $\mu(t,a)$. TB susceptible individuals were at risk of TB infection at a rate $\lambda(t)$ (force of infection). A proportion p and $1-p$ of individuals move, upon TB infection, to the stage of TB latent fast progression (L^F) and the stage of latent slow progression (L^S), respectively. The proportion $p(a)$ differed between children (<15 years old) and adults (≥15 years old). Individuals in L^F and L^S were at risk of TB disease at a rate ω_{LF} and ω_{LS} , respectively. Individuals in L^S were also at risk of reinfection (at a rate $(1-q)\lambda$), but due to prior TB exposure and acquired immunity there was a proportional reduction (*q*) in the susceptibility to TB infection.

TB disease individuals were characterized into the three states of smear-positive pulmonary (I^{SP}), smear-negative pulmonary (I^{SN}), and extra-pulmonary disease (I^{EP}). The parameter $\alpha(a)$ identified the fraction of individuals going into each of these disease states, and differed between children and adults [3]. We considered individuals with the pulmonary TB disease types (I^{SP} and I^{SN}) infectious, but at varying levels. Individuals in the TB disease states could leave their state by TB-related mortality at a rate ξ , by spontaneous natural recovery at a rate ν (i.e. can recover without medical treatment), or by diagnosis and effective treatment at a rate $z\zeta$. Here, ζ is TB treatment rate, and z is the proportion of new TB disease cases that successfully completed treatment (with or without) bacteriologic evidence of success (“cured” or “treatment

completed”). This proportion was derived from seven treatment outcome measures given by the World Health Organization (WHO) [4]:

$$z = \frac{"cure" + "treatment\ completed"}{"cure" + "treatment\ completed" + "death" + "treatment\ \ failure" + default" + transferred" + "not\ evaluated"}.$$

Treated individuals were characterized according to the three TB disease types: T^{SP}, T^{SN}, and T^{EP}. Individuals in the pulmonary treated states (T^{SP} and T^{SN}) were considered infectious, but at varying levels. Individuals in the treated states were assumed at risk of TB reinfection at a rate $(1-q)\lambda$, TB-related mortality at a rate ξ, spontaneous recovery at a rate ν, or successful treatment completion at a rate ψ.

Individuals who are successfully treated, or those who spontaneously recover, enter the recovery stage (R). Recovered TB individuals were assumed at risk of TB reinfection at a rate $(1-q)\lambda$.

The model accommodates (in principle) the risk of developing DM at a rate $RR_{TB\rightarrow DM} \times \beta$ among individuals with current or previous TB disease (I^{SP}, I^{SN}, T^{SP}, T^{SN}, and R; Figure S1 blue line). Here, $RR_{TB\rightarrow DM}$ is the relative risk (RR) of developing DM in the population with a history of TB disease compared to the general population. However, given that our estimates were generated using a conservative approach, we opted not to factor this effect in our analysis since the evidence of this effect is still inconclusive.

S1.2. TB transmission dynamics in presence of DM

Individuals with DM were assumed at higher risk of mortality due to DM complications at a rate $RR_{DM}(a)\mu(t,a)$. Here, RR_{DM} is the RR of mortality in the DM population compared to the general population.

Based on review of existing evidence , DM was assumed to affect 10 different stages of TB natural history and treatment outcomes, denoted as E_1 to E_{10} in the equations in Section S1.3. The summary of the effects and their effect sizes is found in Table 1 of main manuscript and described in details in Section S3.1.

S1.3. Model structure

TB transmission dynamics for the population without DM

TB and DM susceptible (i.e., TB susceptible individuals without DM):

$$\frac{dS(a)}{dt} = \delta(a)\varphi(t)N_{total} + \eta\left[1-\delta(a)\right]S(a-1) - \left[\eta + \lambda(t) + \mu(t,a) + \beta(t,a)\right]S(a)$$

TB latent infection:

$$\begin{aligned}\frac{dL^F(a)}{dt} &= \eta\left[1-\delta(a)\right]L^F(a-1) + p(a)\lambda(t)S(a) + p(a)\left[1-q\right]\lambda(t)L^S(a) + p(a)\left[1-q\right]\lambda(t)\left[R(a) + T^{SP}(a) + T^{SN}(a) + T^{EP}(a)\right]\\ &\quad - \left[\eta + \omega_{LF} + \mu(t,a) + \beta(t,a)\right]L^F(a)\end{aligned}$$

$$\begin{aligned}\frac{dL^S(a)}{dt} &= \eta\left[1-\delta(a)\right]L^S(a-1) + \left[1-p(a)\right]\lambda(t)S(a) + \left[1-p(a)\right]\left[1-q\right]\lambda(t)\left[R(a) + T^{SP}(a) + T^{SN}(a) + T^{EP}(a)\right]\\ &\quad - \left[\eta + p(a)\left[1-q\right]\lambda(t) + \omega_{LS} + \mu(t,a) + \beta(t,a)\right]L^S(a)\end{aligned}$$

TB disease:

$$\frac{dI^{SP}(a)}{dt} = \eta\left[1-\delta(a)\right]I^{SP}(a-1) + \alpha^{LFtoSP}(a)\omega_{LF}L^F(a) + \alpha^{LStoSP}(a)\omega_{LS}L^S(a) - \left[\eta + \xi_{SP} + z\zeta_{SP} + \nu_{SP} + \mu(t,a) + RR_{TB\to DM}\beta(t,a)\right]I^{SP}(a)$$

$$\frac{dI^{SN}(a)}{dt} = \eta\left[1-\delta(a)\right]I^{SN}(a-1) + \alpha^{LFtoSN}(a)\omega_{LF}L^F(a) + \alpha^{LStoSN}(a)\omega_{LS}L^S(a) - \left[\eta + \xi_{SN} + z\zeta_{SN} + \nu_{SN} + \mu(t,a) + RR_{TB\to DM}\beta(t,a)\right]I^{SN}(a)$$

$$\frac{dI^{EP}(a)}{dt} = \eta\left[1-\delta(a)\right]I^{EP}(a-1) + \alpha^{LFtoEP}(a)\omega_{LF}L^F(a) + \alpha^{LStoEP}(a)\omega_{LS}L^S(a) - \left[\eta + \xi_{EP} + z\zeta_{EP} + \nu_{EP} + \mu(t,a) + RR_{TB\to DM}\beta(t,a)\right]I^{EP}(a)$$

Treated TB disease:

$$\frac{dT^{SP}(a)}{dt} = \eta\left[1-\delta(a)\right]T^{SP}(a-1) + z\zeta_{SP}I^{SP}(a) - \left[\eta + \nu_{TSP} + \psi_{TSP} + \left(1-q\right)\lambda(t) + \xi_{TSP} + \mu(t,a) + RR_{TB\rightarrow DM}\beta(t,a)\right]T^{SP}(a)$$

$$\frac{dT^{SN}(a)}{dt} = \eta\left[1-\delta(a)\right]T^{SN}(a-1) + z\zeta_{SN}I^{SN}(a) - \left[\eta + \nu_{TSN} + \psi_{TSN} + \left(1-q\right)\lambda(t) + \xi_{TSN} + \mu(t,a) + RR_{TB\rightarrow DM}\beta(t,a)\right]T^{SN}(a)$$

$$\frac{dT^{EP}(a)}{dt} = \eta\left[1-\delta(a)\right]T^{EP}(a-1) + z\zeta_{EP}I^{EP}(a) - \left[\eta + \nu_{TEP} + \psi_{TEP} + \left(1-q\right)\lambda(t) + \xi_{TEP} + \mu(t,a) + RR_{TB\rightarrow DM}\beta(t,a)\right]T^{EP}(a)$$

Recovered:

$$\begin{aligned}\frac{dR(a)}{dt} &= \eta\left[1-\delta(a)\right]R(a-1) + \underbrace{\psi_{TSP}T^{SP}(a) + \psi_{TSN}T^{SN}(a) + \psi_{TEP}T^{EP}(a)}_{\text{Treatment success}} \\ &+ \underbrace{\nu_{SP}I^{SP}(a) + \nu_{SN}I^{SN}(a) + \nu_{EP}I^{EP}(a) + \nu_{TSP}T^{SP}(a) + \nu_{TSN}T^{SN}(a) + \nu_{TNP}T^{EP}(a)}_{\text{Spontaneous recovery}} \\ &- \left[\eta + \left[1-q\right]\lambda(t) + \mu(t,a) + \beta(t,a)\right]R(a)\end{aligned}$$

TB transmission dynamics for the population with DM (aged ≥ 5 years)

TB susceptible with DM:

$$\frac{dS^{DM}(a)}{dt} = \eta\left[1-\delta(a)\right]S^{DM}(a-1) + \beta(t,a)S(a) - \left[\eta + E_1\lambda(t) + RR_{DM}(a)\mu(t,a)\right]S^{DM}(a)$$

TB latent infection with DM:

$$\begin{aligned}\frac{dL^{F_{DM}}(a)}{dt} &= \eta\left[1-\delta(a)\right]L^{F_{DM}}(a-1) + \beta(t,a)L^{F}(a) + E_2 p(a)E_1\lambda(t)S^{DM}(a) + p(a)E_4\left[1-q\right]\lambda(t)L^{S_{DM}}(a) \\ &+ p(a)E_{10}\left[1-q\right]\lambda(t)\left[T^{SP_{DM}}(a) + T^{SN_{DM}}(a) + T^{NP_{DM}}(a) + R^{DM}(a)\right] - \left[\eta + E_3\omega_{LF} + RR_{DM}(a)\mu(t,a)\right]L^{F_{DM}}(a)\end{aligned}$$

$$\begin{aligned}\frac{dL^{S_{DM}}(a)}{dt} &= \eta\left[1-\delta(a)\right]L^{S_{DM}}(a-1) + \beta(t,a)L^{S}(a) + \left[1-E_2 p(a)\right]E_1\lambda(t)S^{DM}(a) \\ &+ \left[1-p(a)\right]E_{10}\left[1-q\right]\lambda(t)\left[T^{SP_{DM}}(a) + T^{SN_{DM}}(a) + T^{NP_{DM}}(a) + R^{DM}(a)\right] - \left[\eta + E_4 p(a)\left[1-q\right]\lambda(t) + E_3\omega_{LS} + RR_{DM}(a)\mu(t,a)\right]L^{S_{DM}}(a)\end{aligned}$$

TB disease with DM:

$$\frac{dI^{SP_{DM}}(a)}{dt} = \eta\left[1-\delta(a)\right]I^{SP_{DM}}(a-1) + RR_{TB\to DM}\beta(t,a)I^{SP}(a) + \alpha^{(LFtoSP)_{DM}}(a)E_3\omega_{LF}L^{F_{DM}}(a) + \alpha^{(LStoSP)_{DM}}(a)E_3\omega_{LS}L^{S_{DM}}(a)$$
$$-\left[\eta + E_8 z\zeta_{SP} + E_9\nu_{SP} + E_7\xi_{SP} + RR_{DM}(a)\mu(t,a)\right]I^{SP_{DM}}(a)$$

$$\frac{dI^{SN_{DM}}(a)}{dt} = \eta\left[1-\delta(a)\right]I^{SN_{DM}}(a-1) + RR_{TB\to DM}\beta(t,a)I^{SN}(a) + \alpha^{(LFtoSN)_{DM}}(a)E_3\omega_{LF}L^{F_{DM}}(a) + \alpha^{(LStoSN)_{DM}}(a)E_3\omega_{LS}L^{S_{DM}}(a)$$
$$-\left[\eta + E_8 z\zeta_{SN} + E_9\nu_{SN} + E_7\xi_{SN} + RR_{DM}(a)\mu(t,a)\right]I^{SN_{DM}}(a)$$

$$\frac{dI^{EP_{DM}}(a)}{dt} = \eta\left[1-\delta(a)\right]I^{EP_{DM}}(a-1) + RR_{TB\to DM}\beta(t,a)I^{EP}(a) + \alpha^{(LFtoEP)_{DM}}(a)E_3\omega_{LF}L_a^{F_{DM}}(a) + \alpha^{(LStoEP)_{DM}}(a)E_3\omega_{LS}L^{S_{DM}}(a)$$
$$-\left[\eta + E_8 z\zeta_{EP} + E_9\nu_{EP} + E_7\xi_{EP} + RR_{DM}(a)\mu(t,a)\right]I^{EP_{DM}}(a)$$

<u>Treated TB disease with DM:</u>

$$\frac{dT^{SP_{DM}}(a)}{dt} = \eta\left[1-\delta(a)\right]T^{SP_{DM}}(a-1) + RR_{TB\to DM}\beta(t,a)T^{SP}(a) + E_8 z\zeta_{SP}I^{SP_{DM}}(a)$$
$$-\left[\eta + E_9\nu_{TSP} + E_9\psi_{TSP} + E_{10}\left[1-q\right]\lambda(t) + E_7\xi_{TSP} + RR_{DM}(a)\mu(t,a)\right]T^{SP_{DM}}(a)$$

$$\frac{dT^{SN_{DM}}(a)}{dt} = \eta\left[1-\delta(a)\right]T^{SN_{DM}}(a-1) + RR_{TB\to DM}\beta(t,a)T^{SN}(a) + E_8 z\zeta_{SN}I^{SN_{DM}}(a)$$
$$-\left[\eta + E_9\nu_{TSN} + E_9\psi_{TSN} + E_{10}\left[1-q\right]\lambda(t) + E_7\xi_{TSN} + RR_{DM}(a)\mu(t,a)\right]T^{SN_{DM}}(a)$$

$$\frac{dT^{EP_{DM}}(a)}{dt} = \eta\left[1-\delta(a)\right]T^{EP_{DM}}(a-1) + RR_{TB\to DM}\beta(t,a)T^{EP}(a) + E_8 z\zeta_{EP}I^{EP_{DM}}(a)$$
$$-\left[\eta + E_9\nu_{TEP} + E_9\psi_{TEP} + E_{10}\left[1-q\right]\lambda(t) + E_7\xi_{TEP} + RR_{DM}(a)\mu(t,a)\right]T^{EP_{DM}}(a)$$

<u>TB recovered with DM:</u>

$$\frac{dR^{DM}(a)}{dt} = \eta R^{DM}(a-1) + RR_{TB\to DM}\beta(t,a)R(a) + \underbrace{E_9\left[\psi_{TSP}T^{SP_{DM}}(a) + \psi_{TSN}T^{SN_{DM}}(a) + \psi_{TEP}T^{EP_{DM}}(a)\right]}_{\text{Treatment success}}$$
$$+\underbrace{E_9\left[\nu_{SP}I^{SP_{DM}}(a) + \nu_{SN}I^{SN_{DM}}(a) + \nu_{EP}I^{EP_{DM}}(a) + \nu_{TSP}T^{SP_{DM}}(a) + \nu_{TSN}T^{SN_{DM}}(a) + \nu_{TEP}T^{EP_{DM}}(a)\right]}_{\text{Spontaneous recovery}}$$
$$-\left[\eta + E_{10}\left[1-q\right]\lambda(t) + RR_{DM}(a)\mu(t,a)\right]R^{DM}(a)$$

Here, the parameter α for the population with DM was determined according to:

$$\begin{aligned}
&\alpha^{(LFtoSP)_{DM}}(a) = \alpha^{(LStoSP)_{DM}}(a) = E_5^{SP}(a)\alpha^{LFtoSP}(a), \\
&\alpha^{(LFtoSN)_{DM}}(a) = \alpha^{(LStoSN)_{DM}}(a) = E_5^{SN}(a)\alpha^{LFtoSN}(a), \qquad \textbf{(S1)} \\
&\alpha^{(LFtoEP)_{DM}}(a) = \alpha^{(LStoEP)_{DM}}(a) = \alpha^{LFtoEP}(a)
\end{aligned}$$

while,

$$E_5^{SP}(a)=\kappa\frac{\alpha^{(LFtoSP)}(a)+\alpha^{(LFtoSN)}(a)}{\left[\kappa\alpha^{(LFtoSP)}(a)+\kappa'\alpha^{(LFtoSN)}(a)\right]}$$
$$E_5^{SN}(a)=\kappa'\frac{\alpha^{(LFtoSP)}(a)+\alpha^{(LFtoSN)}(a)}{\left[\kappa\alpha^{(LFtoSP)}(a)+\kappa'\alpha^{(LFtoSN)}(a)\right]},\qquad \textbf{(S2)}$$

and

$$\kappa=\frac{P_{DM}^{SP}}{P_{NDM}^{SP}}$$
$$\kappa'=\frac{1-P_{DM}^{SP}}{1-P_{NDM}^{SP}}.\qquad \textbf{(S3)}$$

Here, P_{DM}^{SP} and P_{NDM}^{SP} are the proportions of smear-positive pulmonary TB cases in the DM and non-DM groups, respectively. These proportions were obtained from observational studies discussed in Section S3.1.

S1.4. Demographic parameters

Total number of individuals in the population, N_{total}, was given by:

$$N_{total}=\sum_{a=1}^{20}\begin{pmatrix} S(a)+L^F(a)+L^S(a)+I^{SP}(a)+I^{SN}(a)+I^{EP}(a)+T^{SP}(a)+T^{SN}(a)+T^{EP}(a)+R(a) \\ +S^{DM}(a)+L^{F_{DM}}(a)+L^{S_{DM}}(a)+I^{SP_{DM}}(a)+I^{SN_{DM}}(a)+I^{EP_{DM}}(a)+T^{SP_{DM}}(a)+T^{SN_{DM}}(a)+T^{EP_{DM}}(a)+R^{DM}(a)\end{pmatrix}$$

The population growth rate ($\varphi(t)$) and the natural mortality rate ($\mu(t,a)$) were described by the following functions, providing a robust fit of population growth and age structure in India:

$$\varphi(t)=a_0e^{-\left(\frac{t-t_0}{b_0}\right)^2}$$

and

$$\mu(t,a)=\frac{a_1 e^{-\left(\frac{t-t_1}{b_1}\right)^2}}{\left[1+e^{-b_2(a-a_2)}\right]}$$

Here the parameters a_0, a_1, a_2, t_0, t_1, b_0, b_1, and b_2 were obtained by fitting the model to India's demographic data from the database of the Population Division of the United Nations Department of Economic and Social Affairs [5].

S1.5. TB force of infection and temporal evolution of TB contact rate

Assuming that the mixing between individuals in the population was random, the TB force of infection (λ) was determined by the probability of transmission per respiratory contact (u), the respiratory contact rate within a population (ε), the effect of DM on TB infectiousness (E_6), and the relative infectiousness of individuals with each type of TB disease (whether untreated or treated) compared to the infectiousness of individuals with smear-positive pulmonary TB (h):

$$\lambda(t)=\frac{u\varepsilon(t)\sum_{a=1}^{20}\left(h_{SP}I^{SP}(t,a)+h_{SN}I^{SN}(t,a)+h_{EP}I^{EP}(t,a)+h_{TSP}T^{SP}(t,a)+h_{TSN}T^{SN}(t,a)+h_{TEP}T^{EP}(t,a)\right)}{N_{total}(t,a)}$$
$$+\frac{E_6u\varepsilon(t)\sum_{a=1}^{20}\left(h_{SP_{DM}}I^{SP_{DM}}(t,a)+h_{SN_{DM}}I^{SN_{DM}}(t,a)+h_{EP_{DM}}I^{EP_{DM}}(t,a)+h_{TSP_{DM}}T^{SP_{DM}}(t,a)+h_{TSN_{DM}}T^{SN_{DM}}(t,a)+h_{TEP_{DM}}T^{EP_{DM}}(t,a)\right)}{N_{total}(t,a)}$$

Given the evidence for declining TB incidence in India , a temporal change in ε was incorporated in the model. The temporal variation was characterized through a Wood-Saxon function [6,7]. This function is mathematically designed to describe and characterize transitions. It parameterizes a transition in terms of its scale or strength, smoothness or abruptness, duration, and the turning year [6,7]. Using the Wood-Saxon parameterization, $\varepsilon(t)$ was given by:

$$\varepsilon(t) = \varepsilon_0 \left(1 + \frac{Z}{1 + \exp\left[\left(t - \xi_{Turning} \right) / \xi_{Duration} \right]} \right).$$

Here, ε_0 is the asymptotic value that describes the contact rate well after the transition, Z is the level of change in $\varepsilon(t)$ during the transition from $\varepsilon_0 (1+Z)$ before the transition to ε_0 after the transition, $\xi_{Duration}$ describes the transition duration parameter, and $\xi_{Turning}$ is the turning point year at which the contact rate crosses half way towards its asymptotic value of ε_0. The parameters ε_0, Z, $\xi_{Duration}$, and $\xi_{Turning}$ were obtained by fitting the model to available empirical data on TB-incidence and mortality from the WHO's Global Health Observatory data repository [8].

S1.6. TB treatment rate and temporal evolution of TB case detection rate

Treatment rate in the model depended on TB disease type and was determined using the case detection rates (C_{DetSP}, C_{DetSN}, and C_{DetEP}), TB-related mortality rates (ν_{SP}, ν_{SN}, ν_{EP}), and spontaneous recovery rates (ξ_{SP}, ξ_{SN}, ξ_{EP}):

$$\zeta_{SP} = C_{DetSP}(\nu_{SP} + \xi_{SP}) / (1 - C_{DetSP})$$
$$\zeta_{SN} = C_{DetSN}(\nu_{SN} + \xi_{SN}) / (1 - C_{DetSN})$$
$$\zeta_{EP} = C_{DetEP}(\nu_{EP} + \xi_{EP}) / (1 - C_{DetEP}).$$

Given the evidence for increasing TB case detection in India [9], temporal changes in TB case detection rates were incorporated in the model. Moreover, given the likelihood of underreporting of treatment among TB cases, TB case detection rate for India was derived by fitting the model to TB incidence rate and mortality rate. The temporal variation was parametrized through a logistic function:

$$C_{DetX}(t) = \frac{p_1}{\left[1 + e^{-p_2(t - p_3)}\right]}.$$

Here, the parameters p_1, p_2, and p_3 were obtained by fitting the model to available empirical data on TB-incidence and mortality rates from the WHO's Global Health Observatory data repository [8].

S1.7. DM incidence rate

Given the evidence for increasing DM incidence and prevalence in India [10], the DM incidence rate in the TB-DM model was assumed to be time and age dependent, and was parameterized through a combined Gaussian-logistic function:

$$\beta(t,a) = \frac{c_1 e^{-\left(\frac{t - t_1}{d_1}\right)^2}}{\left[1 + e^{-d_2(a - c_2)}\right]}.$$

Here, c_1, c_2, t_1, d_1, and d_2 are fitting parameters obtained by fitting the TB-DM model to the time series of DM prevalence in India as provided through the International Diabetes Federation [10]. The shape of the age-distribution of DM prevalence was based on the national Indian Council of Medical Research-India Diabetes study [11].

S2. DATA SOURCES

The TB-DM interaction model was parameterized using empirical epidemiological and natural history data from multiple sources.

S.2.1. TB epidemiological and natural history data

The model's parameter values for TB natural history in absence of DM, along with their references, are listed in Table S1.

Table S1. Model assumptions in terms of parameter values.

Symbol	Definition	Parameter value		Sources
		**0-14 years old**	**15+ years old**	
p	Proportion of TB infections entering latent-fast state	5%	15%	[3,12]
α	Proportions of new TB disease cases in each of the three clinical disease categories[#]	$\alpha^{XtoSP}=10\%$<br>$\alpha^{XtoSN}=65\%$<br>$\alpha^{XtoEP}=25\%$	$\alpha^{XtoSP}=50\%$<br>$\alpha^{XtoSN}=40\%$<br>$\alpha^{XtoEP}=10\%$	[13]
q	Fractional reduction in the susceptibility to TB reinfection due to prior exposure to TB	$0.65\ (0.55-0.75)$		[3,14]
ω_{LF}	Progression rate from latency to TB disease for latent-fast progressors (per year)	$0.90\ (0.77-1.04)$		[3]
ω_{LS}	Progression rate from latency to TB disease for latent-slow progressors (per year)	$0.00075\ (0.00064-0.00086)$		[3,14]
ξ	TB disease mortality rate per TB disease category for untreated and treated cases (per year)	$\xi_{SP}=0.25\ (0.21-0.29)$<br>$\xi_{SN}=0.10\ (0.085-0.12)$<br>$\xi_{EP}=0.10\ (0.085-0.12)$<br>$\xi_{TSP}=0.25\ (0.21-0.29)$<br>$\xi_{TSN}=0.10\ (0.085-0.12)$<br>$\xi_{TEP}=0.10\ (0.085-0.12)$		[3,15]
z	Proportion of TB disease cases that are effectively treated	84%		[4]
ν	Spontaneous recovery rate (per year)	$\nu_{SP}=0.10$, $\nu_{SN}=0.10$, $\nu_{EP}=0.10$<br>$\nu_{TSP}=0$, $\nu_{TSN}=0$, $\nu_{TEP}=0$		[3,12,14]
ψ	Rate of successful completion of treatment (per year)	$\psi_{TSP}=2.00\ (1.70-2.30)$<br>$\psi_{TSN}=2.00\ (1.70-2.30)$<br>$\psi_{TEP}=2.00\ (1.70-2.30)$		[16]

u	Transmission probability per respiratory contact	10%	[3]
h	Relative infectiousness for each of the three disease categories and treatment categories with respect to smear-positive pulmonary disease	$h_{SP} = 100\%$<br>$h_{SN} = 25\%$<br>$h_{EP} = 0\%$<br>$h_{TSP} = 13\%$<br>$h_{TSN} = 3.3\%$<br>$h_{TEP} = 0\%$	[3,17,18]
$RR_{DM}^{Age-group}$	Relative risk of mortality in people with DM (per age group) compared to the general population	$RR_{DM}^{0-4} = 1.00$<br>$RR_{DM}^{5-14} = 2.67$<br>$RR_{DM}^{15-29} = 4.83$<br>$RR_{DM}^{30-39} = 4.46$<br>$RR_{DM}^{40-49} = 2.67$<br>$RR_{DM}^{50-59} = 2.19$<br>$RR_{DM}^{60-69} = 1.85$<br>$RR_{DM}^{70-79^{+}} = 1.59$	Calculated based on [19,20]
**Country specific variables**			
N	Total population	For each year per the database of the Population Division of the United Nations Department of Economic and Social Affairs	[5]
φ	Birth rate	Gaussian function	Fitting parameters
μ	Natural mortality rate	Combination of logistic and Gaussian functions	Fitting parameters
C	Case detection rate per TB disease category	Logistic function	Fitting parameters
ε	Respiratory contact rate (per year)	Wood Saxon (logistic function)	Fitting parameters
β	DM incidence rate (per year)	Combination of logistic and Gaussian functions	Fitting parameters

#The three clinical categories are smear-positive pulmonary (SP), smear-negative pulmonary (SN), and extra-pulmonary (EP) tuberculosis. *X* is latent slow (LS) or latent fast (LF).

S2.2. Parametrization of DM-on-TB effects

We incorporated seven out of ten potential DM effects on TB's natural history and treatment outcomes. These are, along with their parameter values, summarized in Table 1 of main text. A brief justification and summary of the evidence for each parameter can be found below. Further details can be found in reference [1].

Effect 1-Susceptibility: DM increases susceptibility to TB infection

Supported by existing evidence [21,22], and based on a recent population-based cross sectional study using the 2011-12 National Health and Nutrition Examination Survey (NHANES) data [23], a 1.5 (95% confidence interval (CI): 1.0-2.2) increased risk of TB infection for individuals with DM, compared to individuals without DM, was incorporated in the model.

Effect 2-Fast progression: DM increases the proportion of TB infections entering latent-fast state as opposed to latent-slow state

A recent meta-analysis of all available study designs (N=44) and four prospective cohort studies found that DM was associated with a 2.00–fold (95% CI: 1.78-2.24) and a 3.59-fold (95% CI: 2.25–5.73) increased risk of TB disease, respectively.[24] However, it is not possible to determine from these studies whether DM increases *i*) the proportion of TB infections entering latent-fast state as opposed to latent-slow state (*Effect 2-Fast progression*), or *ii*) reactivation of latent slow TB cases (*Effect 3-Reactivation*), or *iii*) reinfection of latent slow TB cases (*Effect 4-Reinfection*), or *iv*) a combination of the three effects.

Given that these potential effects could be acting simultaneously, and their individual effects cannot be disentangled from each other [1], we included in the model only one of these effects (*Effect 2-Fast progression*). The exact effect size for *Effect 2-Fast progression* was estimated by fitting the model outcome to the more conservative pooled association of 2.00 (based on all studies) [24]. The model was also fitted to the pooled hazard ratio of 3.59 (based only on the prospective cohort studies) [24] in a sensitivity analysis. No effect sizes were assumed for *Effect 3-Reactivation* and *Effect 4-Reinfection*, as the impacts of these on TB natural history is presumably implicitly captured by *Effect 2-Fast progression*.

Only two studies estimated the age-specific relative risks (RRs) of the effect of DM on TB disease, and these demonstrated a decrease in the RR with age [25,26]. Among DM individuals,

the RR of TB disease was estimated at 7.79 among those aged 20–29, 9.98 among those aged 30–39, 4.72 among those aged 40–49, 2.30 among those aged 50–59, and 1.76 among those aged 60+ [25]. The age-specific RRs of the effect of DM on TB disease [25] were incorporated in the model (as opposed to the overall effect regardless of age), but only in sensitivity analysis. When this was done, the age effects reported in reference [25] were scaled down to reach the two-fold overall RR of the effect of DM on TB disease in the total population (as determined in the meta-analysis based on all studies [24]).

Effect 5-Smear positivity: DM increases the proportion of new pulmonary TB disease cases going to smear-positive as opposed to smear-negative

Based on current evidence [27,28], the proportion of individuals with extra-pulmonary TB as opposed to pulmonary (PTB) in the model was assumed not to differ based on DM status. However, studies have demonstrated that individuals with concurrent PTB and DM were more likely to be sputum acid-fast bacilli (AFB) smear-positive (i.e. have SP-PTB) as opposed to smear-negative (SN-PTB) [29-44]. Based on this evidence, the fraction of individuals who develop SP-PTB as opposed to SN-PTB was assumed to differ by DM status. These fractions were estimated using the pooled mean proportions (out of all PTB cases) by DM status, with the pooling done using a DerSimonian-Laird random-effects model [45] (Figure S2).

Figure S2. Forest plots presenting the outcomes of the pooled mean proportion of smear-positive pulmonary tuberculosis in different populations (A) without diabetes mellitus and (B) with concurrent diabetes mellitus.

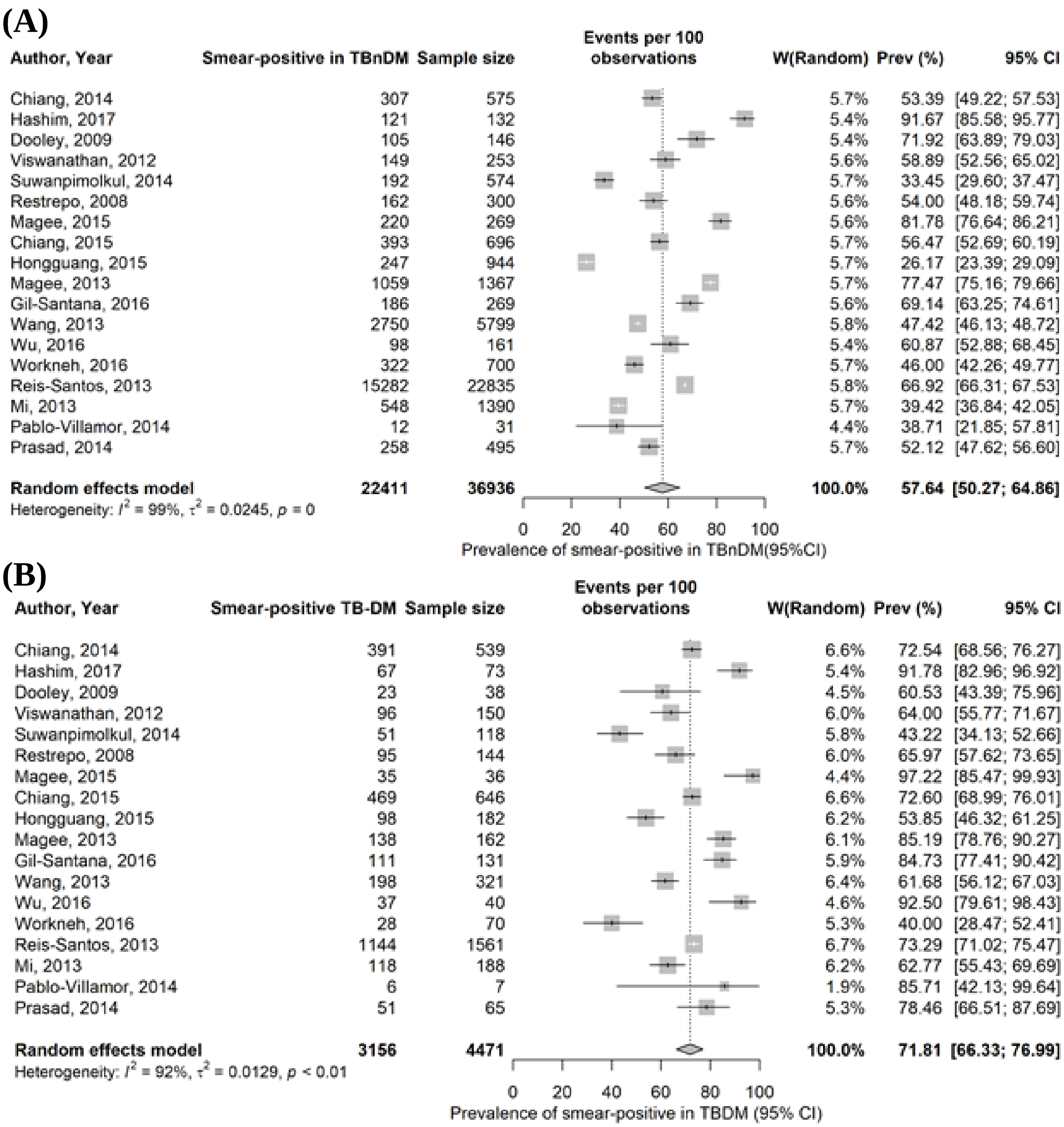

Accordingly using Equations S1-S3 in Section S1.3, for adults upon progressing to TB disease from both L^S and L^F, 63.1% of those with DM will develop SP-PTB compared to 50.0% of those without DM; 26.9% of those with DM will develop SN-PTB compared to 40.0% of those without DM. For children, upon progressing to TB disease from both L^S and L^F, 16.8% of those

with DM will develop SP-PTB compared to 10.0% of those without DM; 58.2% of those with DM will be develop SN-PTB compared to 65.0% of those without DM. The proportions without DM (for adults and children) were based on earlier work [3].

Effect 6-Disease infectiousness: DM increases the infectiousness of PTB (SP-PTB and SN-PTB) for untreated and treated TB disease cases

DM was found to be an independent risk factor associated with increased *M. tuberculosis* bacterial load (based on AFB sputum smear examination) [18,28,29,31,46-48]. Based on this evidence, this effect was incorporated in the model by increasing TB infectiousness among those with concurrent PTB and DM. First, TB infectiousness was assumed to be linearly proportional to *M. tuberculosis* bacterial load. Second, the ratio of TB bacterial load between concurrent TB and DM and TB with no DM individuals was assumed to be equal to the ratio of infectiousness between concurrent TB and DM and TB with no DM individuals. We used available studies to estimate a weighted average of the ratio of bacterial load between concurrent TB and DM and TB with no DM. Accordingly, infectiousness of TB among those with DM was assumed to increase by 1.46-fold compared to those without DM.

Effect 7-TB mortality: DM increases the hazard of TB-related mortality for TB disease cases

Evidence supports an association between DM and TB-related mortality [27,31,49-53]. A recent systematic review and meta-analysis estimated a pooled mean crude odds ratio (OR) of 2.11 (95% CI: 1.76–2.51) across 48 studies, and an adjusted pooled mean OR of 4.95 (95% CI: 2.69–9.10) across four studies that appropriately adjusted for confounders [53]. Based on this evidence, an effect size of 2.11 was incorporated in the TB-DM model as part of our conservative approach for estimating the impact of DM on TB, and an effect size of 4.95 was incorporated as part of a sensitivity analysis. We assumed that, among those who have TB

disease, DM affected (relatively) TB mortality rate similarly for those treated and untreated for TB. The OR of 2.11 was converted to a hazard ratio (HR) using the following equation for *Effect 7-TB mortality* for untreated TB-DM cases:

$$\mathrm{E}_7^X = \frac{\mathrm{OR}(E_8 z\zeta_X + E_9^X \nu_X)}{z\zeta_X + \nu_X}.$$

Here, X is *SP*, *SN*, or *EP*, while E_8 and E_9 are the effects of DM on TB cure and recovery, respectively (see below). The OR was also converted to a HR using the following equation for *Effect 7-TB mortality* for treated TB-DM cases:

$$\mathrm{E}_7^X = \frac{\mathrm{OR}\left[E_9^X(\nu_X + \psi_X) + E_{10}(1-q)\lambda\right]}{\nu_X + \psi_X + (1-q)\lambda}.$$

Here, X is *TSP*, *TSN*, or *TEP*, while E_{10} is the effect of DM on TB reinfection after recovery (see below).

<u>*Effect 8-Treatment failure*: DM reduces the proportion of successful treatment</u>

Several studies reported on “cure” and “treatment completed” as well as the other treatment outcome measures to calculate the proportion of successful treatment with and without DM [42,47,54-67]. Pooling the RR across these studies using a DerSimonian-Laird random-effects model [45], the RR for treatment success was 0.96 (95% CI: 0.93–1.00; Figure S3). Because the RR was not statistically significant, this effect was not included in the TB-DM model—the RR for *Effect 8-Treatment failure* was assumed to equal 1.

Figure S3. Forest plot presenting the outcome of the pooled mean crude relative risk of tuberculosis treatment success with or with no DM in different populations.

	DM		non-DM		
Author, Year	***TB cured***	***TB not cured***	***TB cured***	***TB not cured***	***Relative Risk [95% CI]***
Ambrosetti, 1999	22	10	601	136	0.84 [0.67, 1.07]
Ambrosetti, 1999	36	14	656	117	0.85 [0.71, 1.01]
Ambrosetti, 1999	33	7	519	148	1.06 [0.91, 1.23]
Centis, 2000	26	15	693	366	0.97 [0.76, 1.23]
Centis, 2002	28	12	649	203	0.92 [0.75, 1.13]
Mboussa, 2003	17	23	77	15	0.51 [0.35, 0.74]
Singla, 2006	130	7	367	16	0.99 [0.95, 1.03]
Tatar, 2009	70	3	72	6	1.04 [0.96, 1.13]
Sangral, 2012	19	4	213	46	1.00 [0.83, 1.22]
Jiménez-Corona, 2013	308	55	711	136	1.01 [0.96, 1.07]
Sulaiman, 2013	258	84	717	197	0.96 [0.90, 1.03]
K V N., 2013	554	113	1839	287	0.96 [0.92, 1.00]
Reis-Santos, 2013	441	262	12826	4221	0.83 [0.79, 0.88]
Viswanathan, 2014	80	9	115	5	0.94 [0.87, 1.02]
Alo, 2014	47	6	272	60	1.08 [0.97, 1.21]
Viswanathan, 2014	91	5	142	7	0.99 [0.94, 1.05]
Cavanaugh, 2015	93	8	162	12	0.99 [0.92, 1.06]
Random effect model					0.96 [0.93, 1.00]

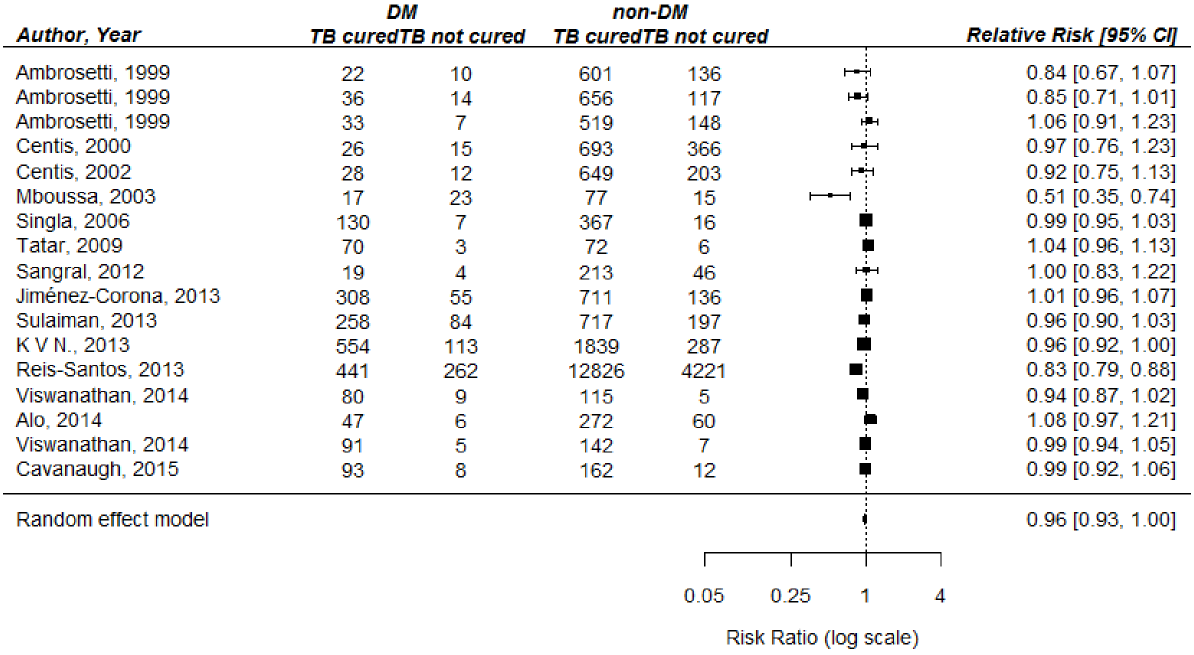

Effect 9-Recovery: DM reduces the rate of TB recovery (prolonging the recovery time) for those who recover naturally or due to treatment

Three studies reported on and compared the number of days it takes to convert from smear positive to smear negative among treated concurrent TB-DM cases and TB-non-DM cases [18,33,61]. The average ratio of the inverse duration (with DM compared to no DM) was pooled across studies by weighting by sample size. The pooled inverse duration ratio was 0.82 implying that DM reduces TB recovery rates (ψ and ν) by 18%.

Effect 10-Cured reinfection: DM increases susceptibility to TB reinfection among those treated or recovered from TB disease

TB reinfection was defined as a subsequent episode of TB disease in a TB patient treated successfully for at least 6 months (i.e. smear or sputum culture was negative at the end of the

treatment period), but developed subsequently active TB. If this new TB episode is due to the same strain as the previous TB episode, this is considered TB relapse, otherwise it is considered TB “recurrence”. Due to the variable definitions for reinfection, recurrence, and relapse in the literature, we opted to define broadly relapse + recurrence as simply “reinfection”.

A recent meta-analysis reported that the risk of TB reinfection is higher (RR of 1.80 95% CI: 1.40-2.30) among those with DM compared to those without DM [53]. This effect size was accordingly incorporated in the TB-DM model.

S3. POPULATION ATTRIBUTABLE FRACTION

The epidemiologic implications of the TB-DM interactions in India were assessed using two (incidence and mortality) "true" population attributable fraction (*PAF*) measures, representing the proportions of all TB disease incidence or mortality that could be prevented if there was no interaction between TB and DM. They were defined as:

$$PAF_{True}^{D} = \frac{D_{TB-DM} - D_{Counter-factual}}{D_{TB-DM}}.$$

Here, D indicates the epidemiological measure of incidence or mortality. D_{TB-DM} is the measure in a scenario where there is a biological synergy between TB and DM, while $D_{Counter-factual}$ is the measure in a counter-factual scenario where the biological synergy between TB and DM is absent.

ADDITIONAL FIGURES

Figure S4. India demographics. (A) Estimated population size between 1980-2050, compared to the projections by the Population Division of the United Nations (UN) Department of Economic and Social Affairs [5]. (B) Estimated population size by age group between 1980-2050, compared to the UN projections.

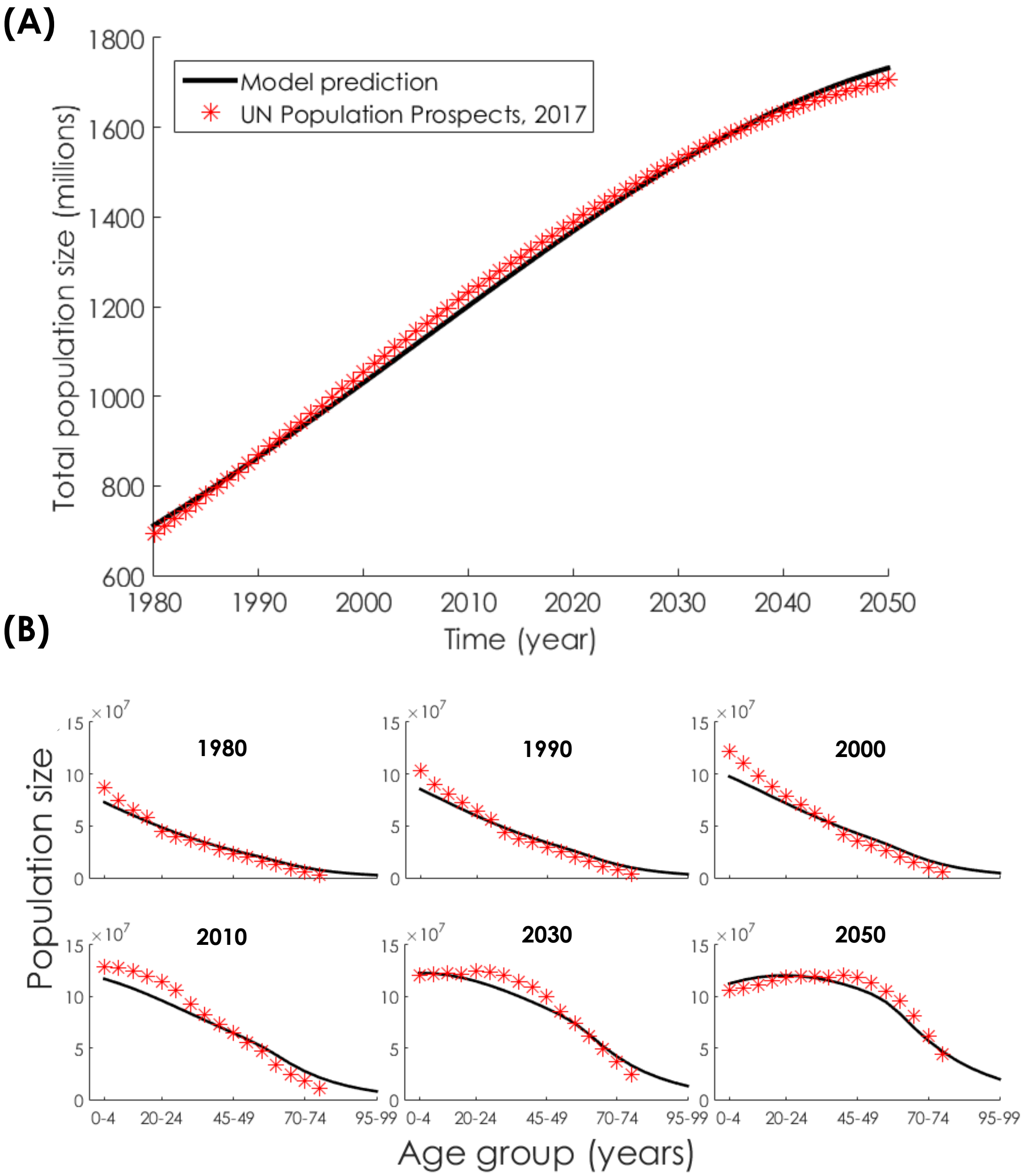

Figure S5. Model projections for the proportion of tuberculosis (TB) disease (A) incident and (B) mortality cases attributed to diabetes mellitus in India at 10 different TB disease incidence rate trajectories. The change in TB incidence rate at 2050, relative to the baseline model scenario, was assumed to range between -50% to +50%.

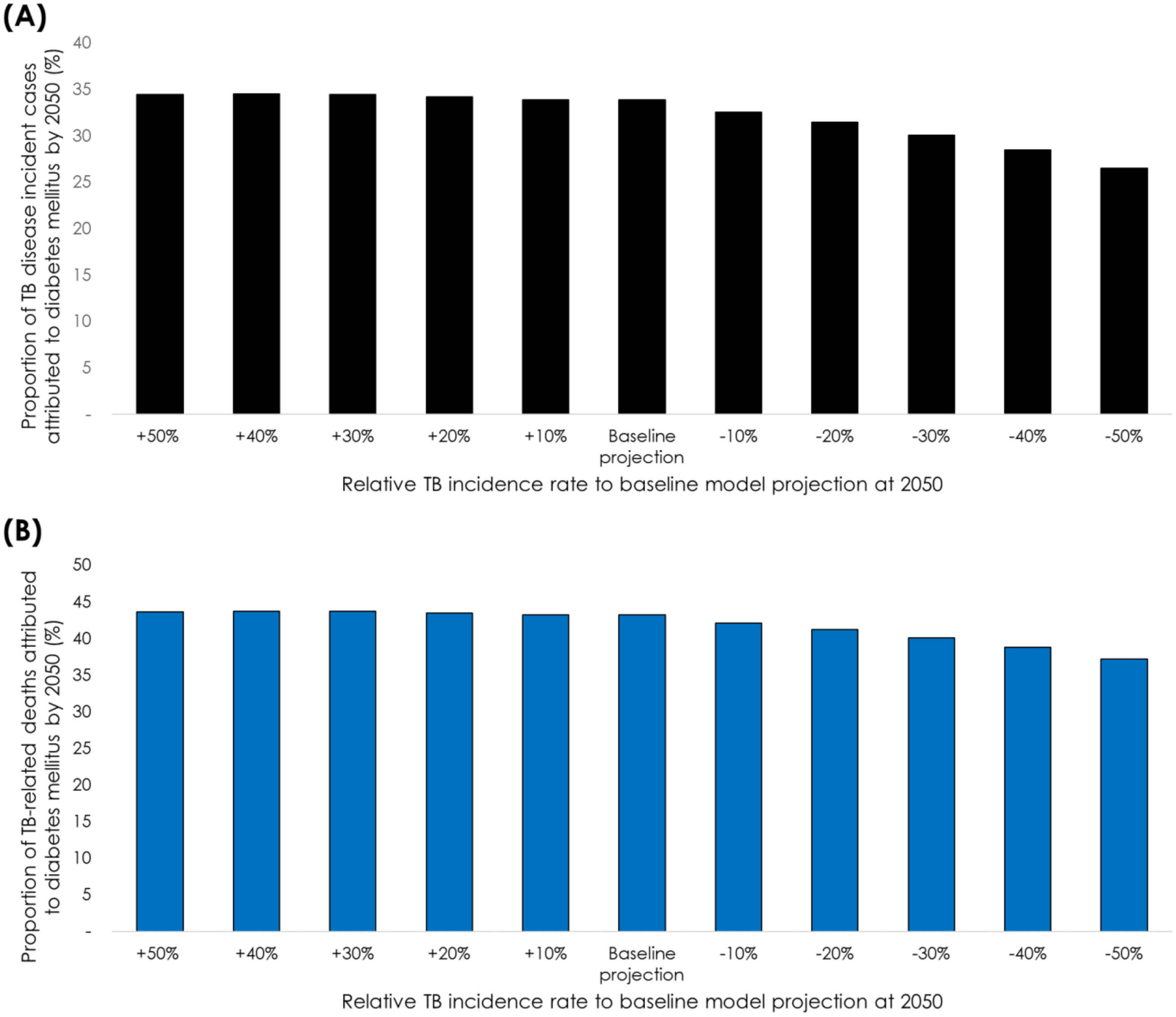

Figure S6. Model predictions for the proportion of tuberculosis (TB) disease incident (solid black line) and mortality (dashes blue line) cases attributed to diabetes mellitus (DM) in India between 1990 and 2050, assuming age-dependency in the proportion of individuals developing latent slow versus latent fast TB infection.

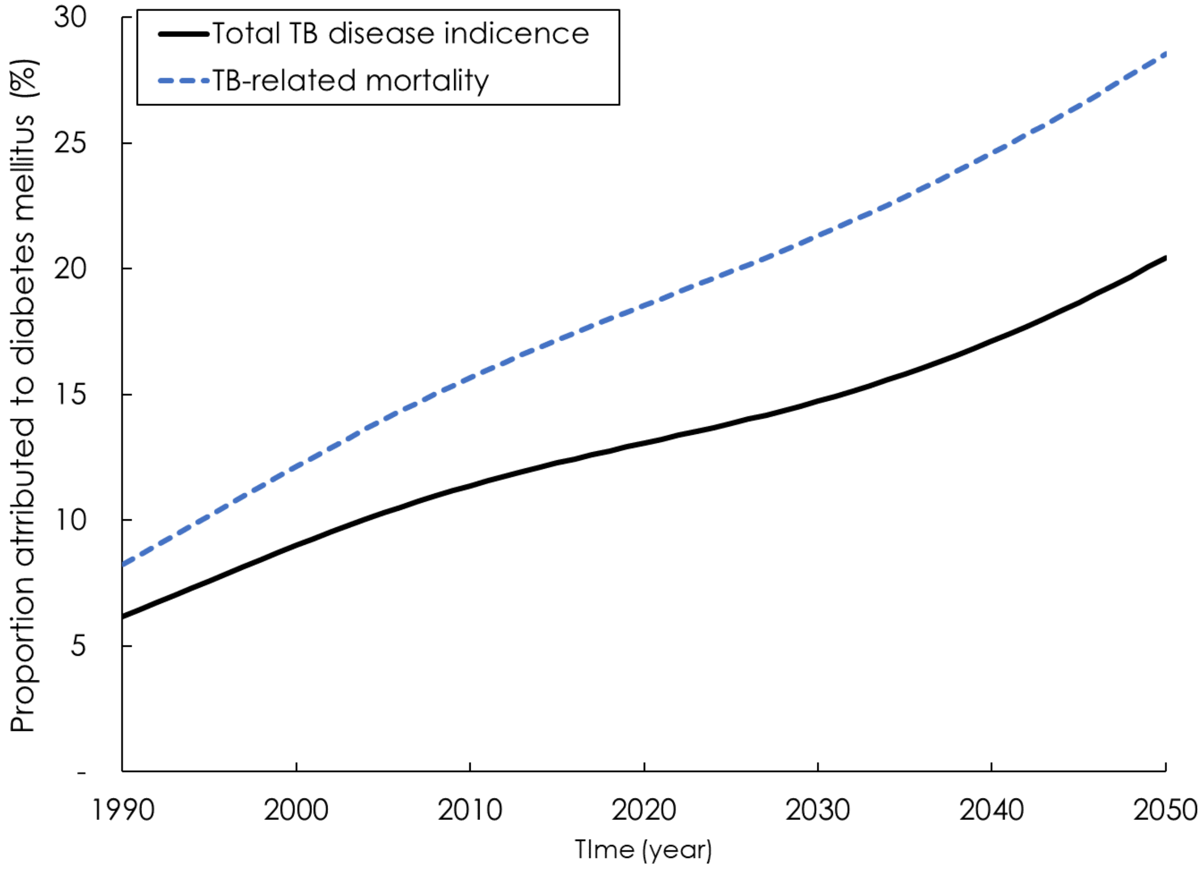

Figure S7. Model predictions for the proportion of tuberculosis (TB) (A) disease incident and (B) mortality cases attributed to diabetes mellitus (DM) in India by 2050, assuming different risk levels of the susceptibility to TB reinfection among individuals *i*) latently infected with TB, *ii*) who successfully completed TB treatment, and *iii*) both latently infected with TB or who successfully completed TB treatment.

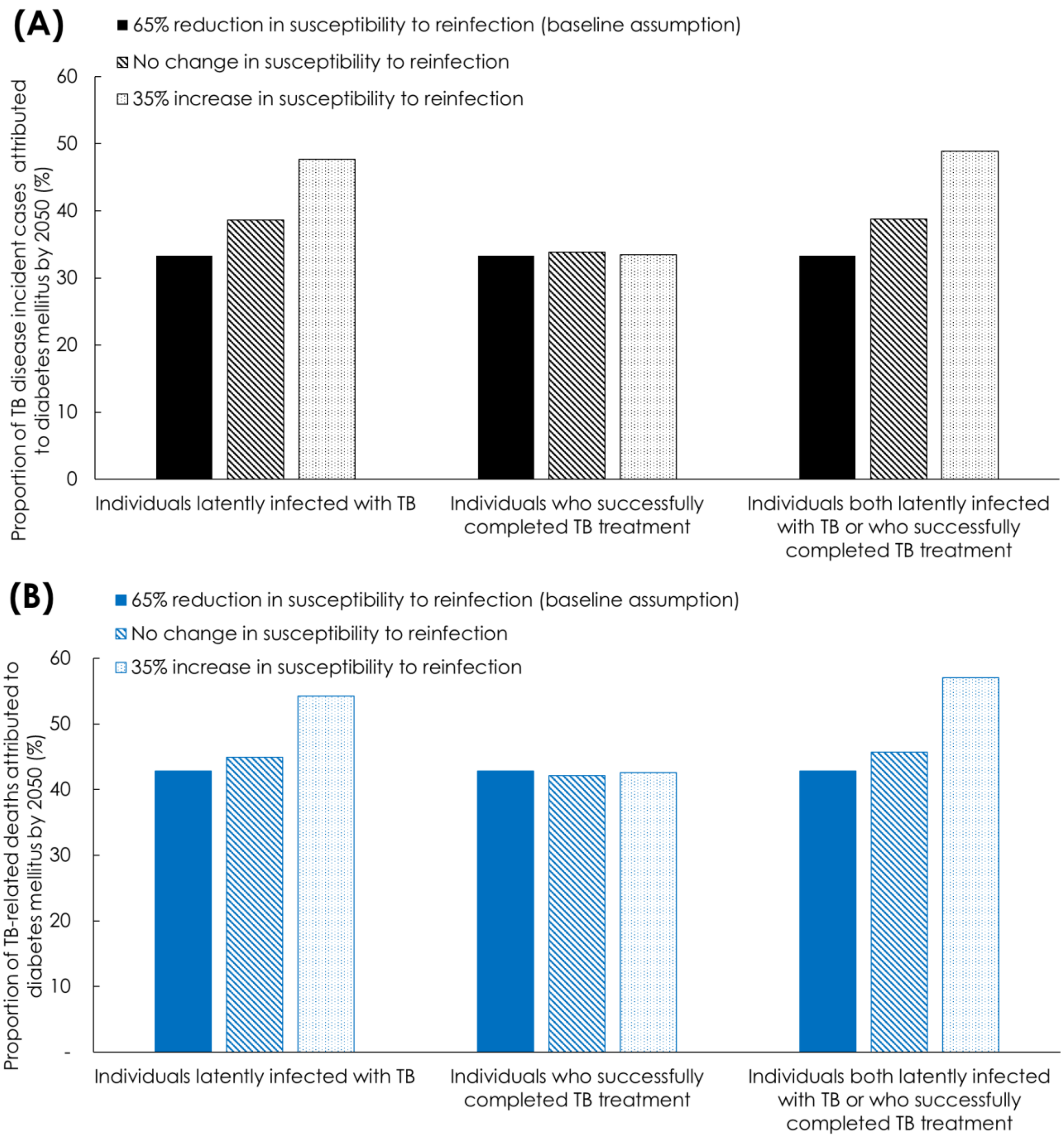

Figure S8. Uncertainty intervals for the proportion of tuberculosis (TB) disease (A) incident and (B) mortality cases attributed to diabetes mellitus in India between 1990 and 2050. The solid red lines represent the mean, while the dashed lines bracket the 95% uncertainty interval.

(A)

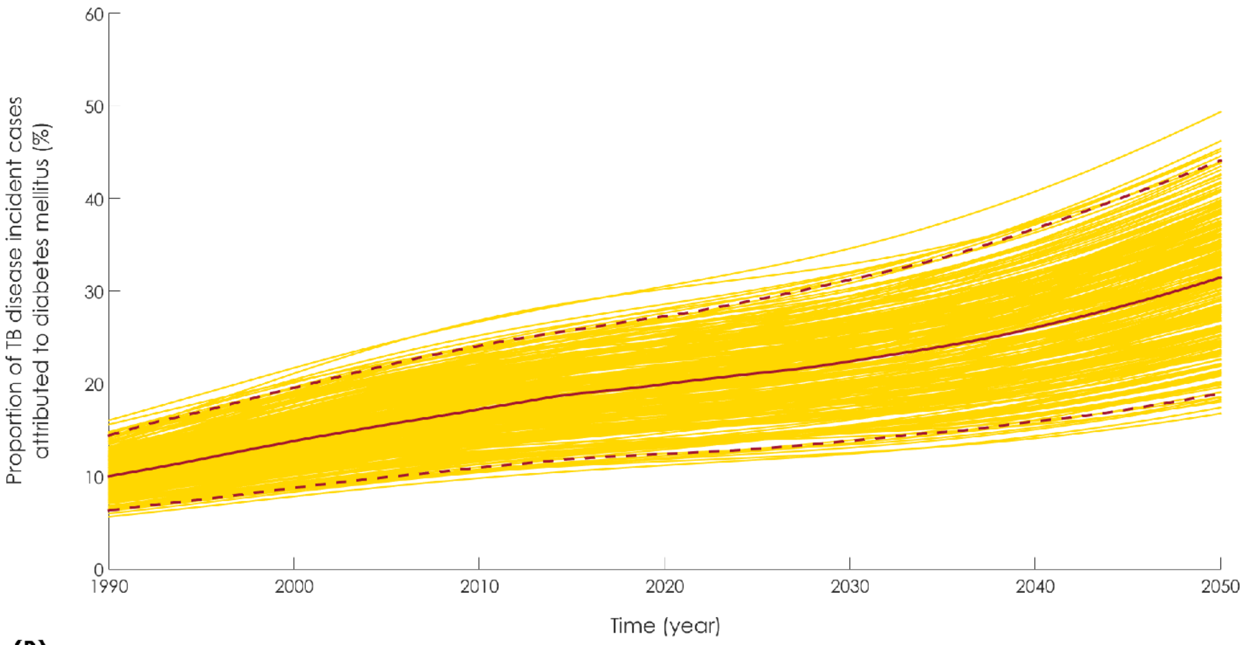

(B)

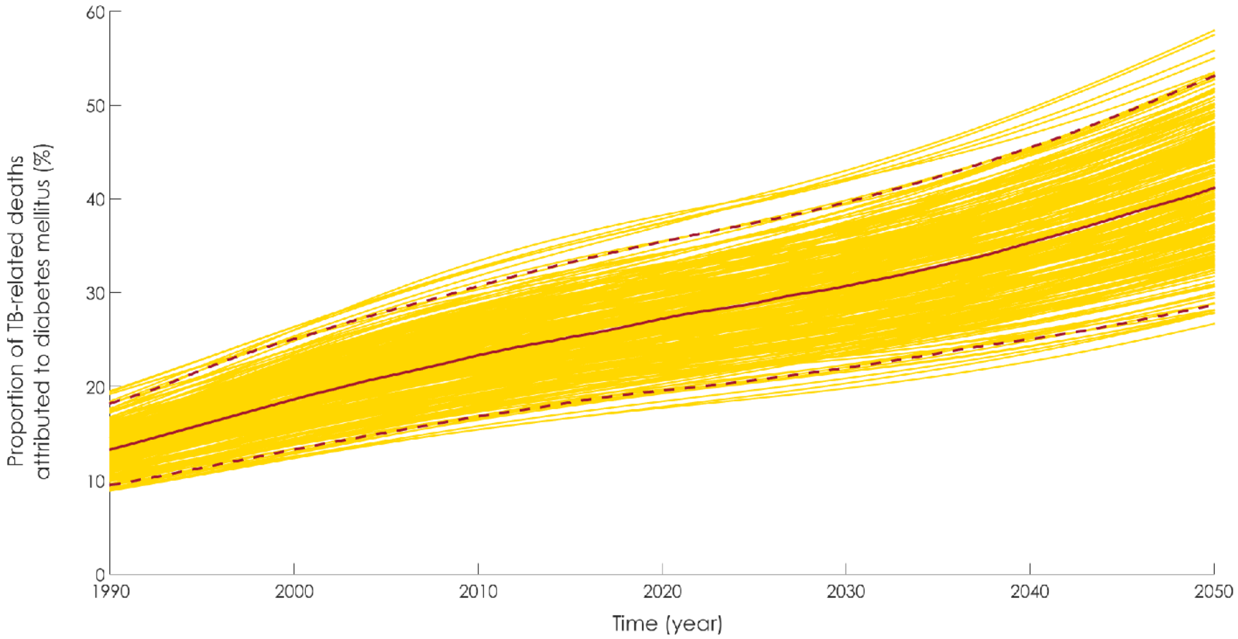

Figure S9. Sensitivity analyses to assess the sensitivity of the proportion of tuberculosis (TB) disease (A) incident and (B) mortality cases attributed to diabetes mellitus in 2050, to variations in the key parameters in the model. Blue bars are based on the lower bound of parameter values (lower bound of the 95% confidence interval; CI) and red bars are based on the upper bound of parameter values (upper bound of the 95% CI; Table 1).

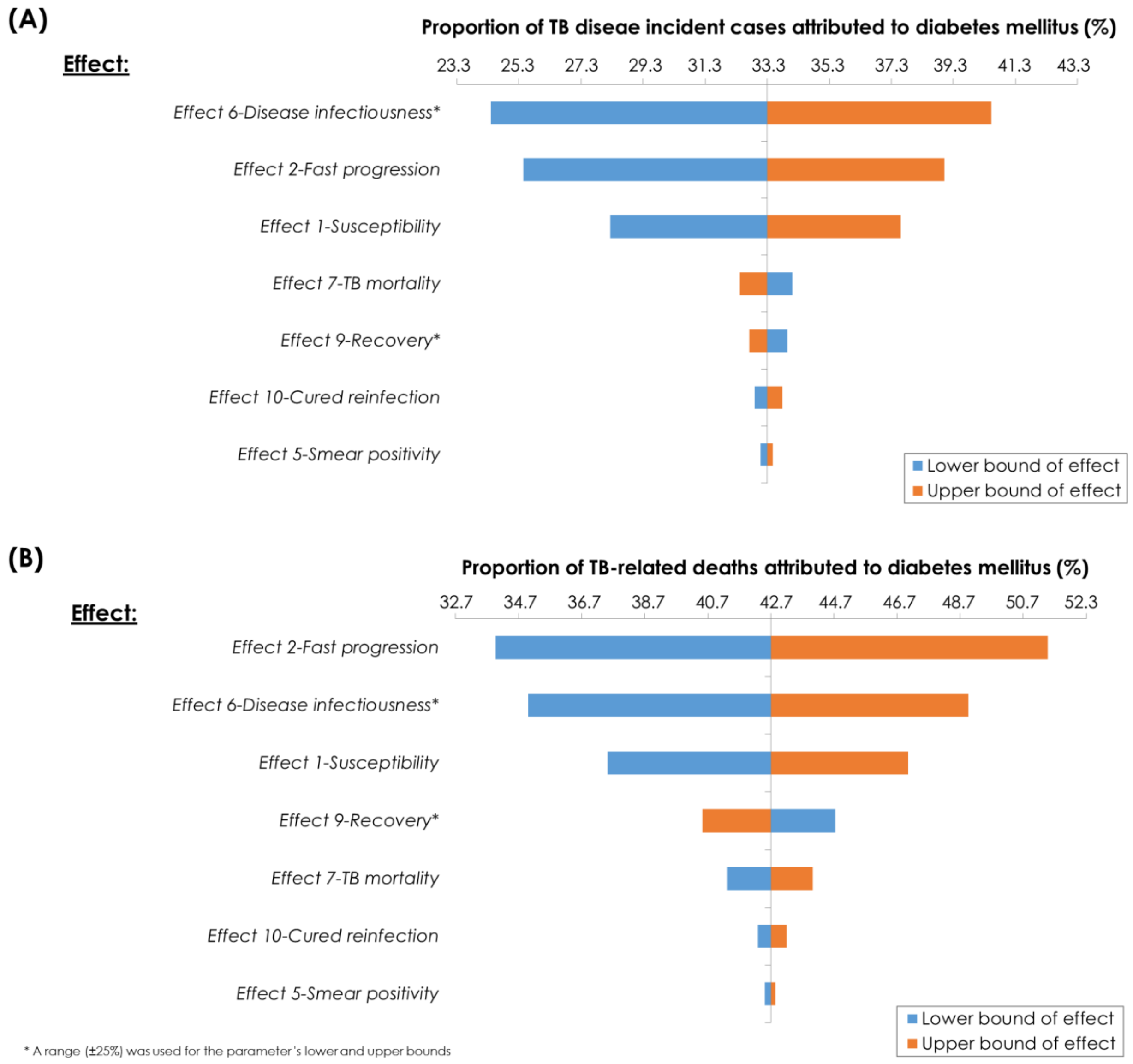

REFERENCES

1 Awad SF, Dargham SR, Omori R, Pearson F, Critchley JA, Abu-Raddad LJ. Analytical Insights into the Impact of Diabetes Mellitus on Tuberculosis Epidemiology. Under review. 2018.

2 The MathWorks, Inc. MATLAB. The language of technical computing. 8.5.0.197613 (R2015a). Natick, MA, USA: ed: The MathWorks, Inc.; 2015.

3 Abu-Raddad LJ, Sabatelli L, Achterberg JT, Sugimoto JD, Longini IM, Jr., Dye C, et al. Epidemiological benefits of more-effective tuberculosis vaccines, drugs, and diagnostics. Proceedings of the National Academy of Sciences of the United States of America. 2009;106:13980-5.

4 World Health Organization. Global Tuberculosis Control Surveillance, Planning, Financing (available at: http://data.unaids.org/pub/report/2008/who2008globaltbreport_en.pdf). Switzerland: World Health Organization, 2008.

5 United Nations Department of Economic and Social Affairs, Population Division, Population Estimates and Projections Section. World population prospects, the 2012 revision. 2012. available: http://esa.un.org/wpp/Excel-Data/population.htm

6 Velicia FJF. On the moments of a Wood Saxon beta distribution. Journal of Physics A: Mathematical and General. 1987.

7 Woods RD, Saxon DS. Diffuse Surface Optical Model for Nucleon-Nuclei Scattering. Physical Review. 1954;95:577-8.

8 World Health Organization. WHO Global Health Observatory Data Repository, (available at: http://apps.who.int/gho/data/node.main). 2017. Accessed.

9 The World Bank. Tuberculosis case detection rate in India (%, all forms) (Available at: https://data.worldbank.org/indicator/SH.TBS.DTEC.ZS?locations=IN&year_high_desc=false; Accessed November, 2017). 2015.

10 International Diabetes Federation. IDF Diabetes Atlas. 7th edition. Brussels, Belgium (Available at:http://www.diabetesatlas.org; accessed on September 2016). 2016.

11 Anjana RM, Deepa M, Pradeepa R, Mahanta J, Narain K, Das HK, et al. Prevalence of diabetes and prediabetes in 15 states of India: results from the ICMR-INDIAB population-based cross-sectional study. The lancet Diabetes & endocrinology. 2017;5:585-96.

12 Dye C, Garnett GP, Sleeman K, Williams BG. Prospects for worldwide tuberculosis control under the WHO DOTS strategy. Directly observed short-course therapy. Lancet. 1998;352:1886-91.

13 Barnett GD, Styblo K. Bacteriological and X-ray status of tuberculosis following primary infection acquired during adolescence or later. Bulletin of the International Union Against Tuberculosis 52:5-16. 1977.

14 Dye C, Williams BG. Eliminating human tuberculosis in the twenty-first century. J R Soc Interface. 2008;5:653-62.

15 Springett VH. Ten-year results during the introduction of chemotherapy for tuberculosis. Tubercle. 1971;52:73-87.

16 World Health Organization. Treatment of tuberculosis: guidelines – 4th ed. (Available at: http://apps.who.int/iris/bitstream/10665/44165/1/9789241547833_eng.pdf, accessed Nov. 2018). 2010.

17 Small PM, Hopewell PC, Singh SP, Paz A, Parsonnet J, Ruston DC, et al. The epidemiology of tuberculosis in San Francisco. A population-based study using conventional and molecular methods. The New England journal of medicine. 1994;330:1703-9.

18 Chang JT, Dou HY, Yen CL, Wu YH, Huang RM, Lin HJ, et al. Effect of type 2 diabetes mellitus on the clinical severity and treatment outcome in patients with pulmonary tuberculosis: a potential

role in the emergence of multidrug-resistance. Journal of the Formosan Medical Association = Taiwan yi zhi. 2011;110:372-81.
19 Nakagami T, Group DS. Hyperglycaemia and mortality from all causes and from cardiovascular disease in five populations of Asian origin. Diabetologia. 2004;47:385-94.
20 International Diabetes Federation. IDF Diabetes Atlas. 3th edition. Brussels, Belgium (available at: https://www.idf.org/sites/default/files/Diabetes-Atlas-3rd-edition.pdf; accessed on December 2015). 2006.
21 Restrepo BI, Schlesinger LS. Impact of diabetes on the natural history of tuberculosis. Diabetes research and clinical practice. 2014;106:191-9.
22 Lee MR, Huang YP, Kuo YT, Luo CH, Shih YJ, Shu CC, et al. Diabetes Mellitus and Latent Tuberculosis Infection: A Systemic Review and Metaanalysis. Clinical infectious diseases : an official publication of the Infectious Diseases Society of America. 2017;64:719-27.
23 Martinez L, Zhu L, Castellanos ME, Liu Q, Chen C, Hallowell BD, et al. Glycemic Control and the Prevalence of Tuberculosis Infection: A Population-based Observational Study. Clinical infectious diseases : an official publication of the Infectious Diseases Society of America. 2017.
24 Al-Rifai RH, Pearson F, Critchley JA, Abu-Raddad LJ. Association between diabetes mellitus and active tuberculosis: A systematic review and meta-analysis. PLoS One. 2017;12:e0187967.
25 Kim SJ, Hong YP, Lew WJ, Yang SC, Lee EG. Incidence of pulmonary tuberculosis among diabetics. Tubercle and Lung Disease. 1995;76:529-33.
26 Ponce-De-Leon A, Garcia-Garcia Md Mde L, Garcia-Sancho MC, Gomez-Perez FJ, Valdespino-Gomez JL, Olaiz-Fernandez G, et al. Tuberculosis and diabetes in southern Mexico. Diabetes care. 2004;27:1584-90.
27 Magee MJ, Foote M, Maggio DM, Howards PP, Narayan KM, Blumberg HM, et al. Diabetes mellitus and risk of all-cause mortality among patients with tuberculosis in the state of Georgia, 2009-2012. Annals of epidemiology. 2014;24:369-75.
28 Duangrithi D, Thanachartwet V, Desakorn V, Jitruckthai P, Phojanamongkolkij K, Rienthong S, et al. Impact of diabetes mellitus on clinical parameters and treatment outcomes of newly diagnosed pulmonary tuberculosis patients in Thailand. International journal of clinical practice. 2013;67:1199-209.
29 Chiang CY, Bai KJ, Lin HH, Chien ST, Lee JJ, Enarson DA, et al. The Influence of Diabetes, Glycemic Control, and Diabetes-Related Comorbidities on Pulmonary Tuberculosis. PLoS ONE. 2015;10:e0121698.
30 Chiang CY, Lee JJ, Chien ST, Enarson DA, Chang YC, Chen YT, et al. Glycemic control and radiographic manifestations of tuberculosis in diabetic patients. PLoS One. 2014;9:e93397.
31 Dooley KE, Tang T, Golub JE, Dorman SE, Cronin W. Impact of Diabetes Mellitus on Treatment Outcomes of Patients with Active Tuberculosis. The American Journal of Tropical Medicine and Hygiene. 2009;80:634-9.
32 Viswanathan V, Kumpatla S, Aravindalochanan V, Rajan R, Chinnasamy C, Srinivasan R, et al. Prevalence of diabetes and pre-diabetes and associated risk factors among tuberculosis patients in India. PLoS One. 2012;7:e41367.
33 Suwanpimolkul G, Grinsdale JA, Jarlsberg LG, Higashi J, Osmond DH, Hopewell PC, et al. Association between diabetes mellitus and tuberculosis in United States-born and foreign-born populations in San Francisco. PLoS One. 2014;9:e114442.
34 Restrepo BI, Fisher-Hoch SP, Smith B, Jeon S, Rahbar MH, McCormick JB, et al. Mycobacterial clearance from sputum is delayed during the first phase of treatment in patients with diabetes. The American journal of tropical medicine and hygiene. 2008;79:541-4.

35 Magee M, Kempker R, Kipiani M, Gandhi N, Darchia L, Tukvadze N, et al. Diabetes mellitus is associated with cavities, smear grade, and multidrug-resistant tuberculosis in Georgia. The International Journal of Tuberculosis and Lung Disease. 2015;19:685-92.

36 Hongguang C, Min L, Shiwen J, Fanghui G, Shaoping H, Tiejie G, et al. Impact of diabetes on clinical presentation and treatment outcome of pulmonary tuberculosis in Beijing. Epidemiology & Infection. 2015;143:150-6.

37 Magee MJ, Bloss E, Shin SS, Contreras C, Huaman HA, Ticona JC, et al. Clinical characteristics, drug resistance, and treatment outcomes among tuberculosis patients with diabetes in Peru. Int J Infect Dis. 2013;17:e404-12.

38 Gil-Santana L, Almeida-Junior JL, Oliveira CA, Hickson LS, Daltro C, Castro S, et al. Diabetes Is Associated with Worse Clinical Presentation in Tuberculosis Patients from Brazil: A Retrospective Cohort Study. PLoS One. 2016;11:e0146876.

39 Wang Q, Ma A, Han X, Zhao S, Cai J, Ma Y, et al. Prevalence of type 2 diabetes among newly detected pulmonary tuberculosis patients in China: a community based cohort study. PLoS One. 2013;8:e82660.

40 Wu Z, Guo J, Huang Y, Cai E, Zhang X, Pan Q, et al. Diabetes mellitus in patients with pulmonary tuberculosis in an aging population in Shanghai, China: Prevalence, clinical characteristics and outcomes. Journal of diabetes and its complications. 2016;30:237-41.

41 Workneh MH, Bjune GA, Yimer SA. Diabetes mellitus is associated with increased mortality during tuberculosis treatment: a prospective cohort study among tuberculosis patients in South-Eastern Amahra Region, Ethiopia. Infect Dis Poverty. 2016;5:22.

42 Reis-Santos B, Locatelli R, Horta BL, Faerstein E, Sanchez MN, Riley LW, et al. Socio-demographic and clinical differences in subjects with tuberculosis with and without diabetes mellitus in Brazil--a multivariate analysis. PLoS One. 2013;8:e62604.

43 Mi F, Tan S, Liang L, Harries AD, Hinderaker SG, Lin Y, et al. Diabetes mellitus and tuberculosis: pattern of tuberculosis, two-month smear conversion and treatment outcomes in Guangzhou, China. Tropical medicine & international health : TM & IH. 2013;18:1379-85.

44 Prasad P, Gounder S, Varman S, Viney K. Sputum smear conversion and treatment outcomes for tuberculosis patients with and without diabetes in Fiji. Public Health Action. 2014;4:159-63.

45 Borenstein M, Hedges LV, Higgins J, Rothstein HR. References: Wiley Online Library; 2009.

46 John NN, George JM, Narmadha M. Study on the prevalence and incidence rates of diabetes mellitus in tuberculosis. International Journal of Pharmaceutical, Chemical & Biological Sciences. 2017;7:401-6.

47 Singla R, Khan N, Al-Sharif N, Ai-Sayegh MO, Shaikh MA, Osman MM. Influence of diabetes on manifestations and treatment outcome of pulmonary TB patients. The international journal of tuberculosis and lung disease : the official journal of the International Union against Tuberculosis and Lung Disease. 2006;10:74-9.

48 Restrepo BI, Fisher-Hoch SP, Crespo JG, Whitney E, Perez A, Smith B, et al. Type 2 diabetes and tuberculosis in a dynamic bi-national border population. Epidemiology and infection. 2007;135:483-91.

49 Wang CS, Yang CJ, Chen HC, Chuang SH, Chong IW, Hwang JJ, et al. Impact of type 2 diabetes on manifestations and treatment outcome of pulmonary tuberculosis. Epidemiology and infection. 2009;137:203-10.

50 Wang JY, Lee LN, Hsueh PR. Factors changing the manifestation of pulmonary tuberculosis. The international journal of tuberculosis and lung disease : the official journal of the International Union against Tuberculosis and Lung Disease. 2005;9:777-83.

51 Baker MA, Harries AD, Jeon CY, Hart JE, Kapur A, Lönnroth K, et al. The impact of diabetes on tuberculosis treatment outcomes: A systematic review. BMC Medicine. 2011;9:81-.

52 Faurholt-Jepsen D, Range N, PrayGod G, Jeremiah K, Faurholt-Jepsen M, Aabye MG, et al. Diabetes is a strong predictor of mortality during tuberculosis treatment: a prospective cohort study among tuberculosis patients from Mwanza, Tanzania. Tropical Medicine & International Health. 2013;18:822-9.
53 Huangfu P, Ugarte-Gil C, Golub J, Pearson F, Critchley J. The effects of diabetes on tuberculosis treatment outcomes: an updated systematic review and meta-analysis Under Review. 2017.
54 Mboussa J, Monabeka H, Kombo M, Yokolo D, Yoka-Mbio A, Yala F. [Course of pulmonary tuberculosis in diabetics]. Rev Pneumol Clin. 2003;59:39-44.
55 Tatar D, Senol G, Cosar D, Ozacar R, Halilcolar H. Patterns of drug resistance in pulmonary tuberculosis cases in the Izmir district, Turkey. New Microbiol. 2009;32:31-7.
56 Jimenez-Corona ME, Cruz-Hervert LP, Garcia-Garcia L, Ferreyra-Reyes L, Delgado-Sanchez G, Bobadilla-Del-Valle M, et al. Association of diabetes and tuberculosis: impact on treatment and post-treatment outcomes. Thorax. 2013;68:214-20.
57 Sulaiman SA, Khan AH, Muttalif AR, Hassali MA, Ahmad N, Iqubal MS. Impact of diabetes mellitus on treatment outcomes of tuberculosis patients in tertiary care setup. Am J Med Sci. 2013;345:321-5.
58 K VN, Duraisamy K, Balakrishnan S, M S, S JS, Sagili KD, et al. Outcome of tuberculosis treatment in patients with diabetes mellitus treated in the revised national tuberculosis control programme in Malappuram District, Kerala, India. PLoS One. 2013;8:e76275.
59 Viswanathan AA, Gawde NC. Effect of type II diabetes mellitus on treatment outcomes of tuberculosis. Lung India : official organ of Indian Chest Society. 2014;31:244-8.
60 Alo A, Gounder S, Graham SM. Clinical characteristics and treatment outcomes of tuberculosis cases hospitalised in the intensive phase in Fiji. Public Health Action. 2014;4:164-8.
61 Viswanathan V, Vigneswari A, Selvan K, Satyavani K, Rajeswari R, Kapur A. Effect of diabetes on treatment outcome of smear-positive pulmonary tuberculosis--a report from South India. Journal of diabetes and its complications. 2014;28:162-5.
62 Cavanaugh J, Viney K, Kienene T, Harley D, Kelly P, Sleigh A, et al. Effect of diabetes on tuberculosis presentation and outcomes in Kiribati. Tropical Medicine & International Health. 2015.
63 Ambrosetti M, Besozzi G, Codecasa LR, Farris B, Nutini S, Saini L, et al. The Italian AIPO study on tuberculosis treatment results, report 1997. National AIPO "Tuberculosis" Study Group. Monaldi Arch Chest Dis. 1999;54:407-12.
64 Ambrosetti M, Besozzi G, Codecasa LR, Farris B, Nutini S, Saini L, et al. The Italian AIPO study on tuberculosis treatment results, report 1995. National AIPO "Tuberculosis" Study Group. Monaldi Arch Chest Dis. 1999;54:49-54.
65 Ambrosetti M, Besozzi G, Farris B, Nutini S, Saini L, Casali L, et al. The Italian AIPO study on tuberculosis treatment results, report 1996. National AIPO "Tuberculosis" Study Group. Associazione Italiana Pneumologi Ospedalieri. Monaldi Arch Chest Dis. 1999;54:237-41.
66 Centis R, Ianni A, Migliori GB. Evaluation of tuberculosis treatment results in Italy, report 1998. Tuberculosis section of the National AIPO Study Group on Infectious Disease and the SMIRA Group. Monaldi Arch Chest Dis. 2000;55:293-8.
67 Centis R, Migliori GB, Tuberculosis Study Group. National AIPO, Group S, National Tuberculosis Project ISdS. Evaluation of tuberculosis treatment results in Italy, report 1999. Monaldi Arch Chest Dis. 2002;57:297-305.
